# Freshwater Megafauna: Flagships for Freshwater Biodiversity under Threat

**DOI:** 10.1093/biosci/bix099

**Published:** 2017-09-20

**Authors:** Savrina F. Carrizo, Sonja C. Jähnig, Vanessa Bremerich, Jörg Freyhof, Ian Harrison, Fengzhi He, Simone D. Langhans, Klement Tockner, Christiane Zarfl, William Darwall

**Affiliations:** Dr. Sonja Jähnig (sonja.jaehnig@igb-berlin.de) is a group leader, Vanessa Bremerich a technician, Dr. Jörg Freyhof a project leader, Dr. Simone D. Langhans a postdoctoral researcher, and Fengzhi He a doctoral student at the Leibniz-Institute of Freshwater Ecology and Inland Fisheries, in Berlin, Germany; FH is also affiliated with the Institute of Biology at Freie Universität Berlin. Dr. Savrina F. Carrizo was a program officer with the International Union for Conservation of Nature (IUCN) Global Species Programme's Freshwater Biodiversity Unit at the time of this research. Dr. Ian Harrison is working for the IUCN Freshwater Fish Specialist Group, in Flagstaff, Arizona. Professor Klement Tockner currently serves as the president of the Austrian Science Fund, in Vienna. Dr. Christiane Zarfl is a junior professor at the Center for Applied Geosciences at Eberhard Karls Universität Tübingen, in Germany. Dr. William Darwall is the head of the IUCN Global Species Programme's Freshwater Biodiversity Unit, in Cambridge, United Kingdom.

**Keywords:** biodiversity, flagship species, freshwater conservation, freshwater megafauna, umbrella species

## Abstract

Freshwater biodiversity is highly threatened and is decreasing more rapidly than its terrestrial or marine counterparts; however, freshwaters receive less attention and conservation investment than other ecosystems do. The diverse group of freshwater megafauna, including iconic species such as sturgeons, river dolphins, and turtles, could, if promoted, provide a valuable tool to raise awareness and funding for conservation. We found that freshwater megafauna inhabit every continent except Antarctica, with South America, Central Africa, and South and Southeast Asia being particularly species rich. Freshwater megafauna co-occur with up to 93% of mapped overall freshwater biodiversity. Fifty-eight percent of the 132 megafauna species included in the study are threatened, with 84% of their collective range falling outside of protected areas. Of all threatened freshwater species, 83% are found within the megafauna range, revealing the megafauna's capacity as flagship and umbrella species for fostering freshwater conservation.

**Freshwater ecosystems cover less than one percent** of the planet, but they are among the most diverse and dynamic systems globally (Strayer and Dudgeon 2010). They provide important functions and services such as water purification, carbon sequestration, and flood regulation, thereby supporting human well-being (Russi et al. 2013). At the same time, freshwaters are among the most threatened ecosystems worldwide. They continue to be degraded rapidly, and biodiversity is lost through human activities at unprecedented rates (Davidson 2014, WWF 2016). Indeed, one in three freshwater species is already threatened (IUCN 2016b), and populations are declining faster than in marine or terrestrial realms (Dudgeon et al. 2006, WWF 2016).

Despite their critical state, freshwaters and their unique diversity remain largely overlooked by the general public and within environmental policy (Cooke et al. 2013). Therefore, rivers, lakes, and ground waters receive less conservation investments than most other ecosystems do (Darwall et al. 2011). The reasons for this investment gap are manifold: For example, far less conservation research has focused on freshwater than on terrestrial ecosystems (Di Marco et al. 2017), which subsequently influences the allocation of conservation funds (Donaldson et al. 2016). At the same time, the hidden nature of freshwater organisms leads to a lack of ­public awareness for them. Additionally, it leads to shifting baselines in public perception of freshwater biodiversity (Turvey et al. 2010), since we are often unaware of biodiversity declines that happened in the past (Humphries and Winemiller 2009).

Terrestrial and marine megafauna species, such as rhinos, elephants, tigers, and whales, have been successfully used as flagship species, gaining strong public attention for decades (Caro and O’Doherty 1999, Hooker and Gerber 2004, Caro 2010, Verissimo et al. 2011). Consequently, these species are widely targeted for conservation actions at regional to global scales (Sodhi et al. 2011), and they continuously attract media attention and conservation funding (GMFER 2016, Price 2016). Freshwater megafauna—such as the beluga, or European sturgeon (*Huso huso)*; large hippo (Hippopotamus amphibius); or the Nile crocodile (Crocodylus niloticus)—are also large in size and spectacular in appearance ­(figure 1). Such impressive species may help generate public interest for the “hidden” freshwater biodiversity, too.

**Figure 1.**
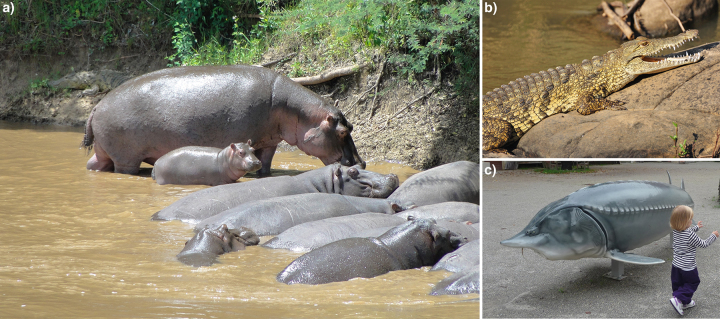
Charismatic freshwater megafauna species. Left to right: (a) large hippo (Hippopotamus amphibius); (b) Nile crocodile (Crocodylus niloticus); (c) model beluga sturgeon (Huso huso). Photographs: Peter Haase, F. David Carmona, and William Darwall.

In this article, we demonstrate the potential for large-bodied freshwater species to be employed as flagship and/or umbrella species promoting the urgent need for freshwater conservation. First, we provide a synoptic and spatially explicit assessment of the distribution and conservation status of global freshwater megafauna. As a proxy for understanding current efforts to conserve freshwater ecosystems, we quantify the spatial extent to which protected areas coincide with the geographic distributions of freshwater megafauna. Second, we investigate the potential conservation umbrella effects of freshwater megafauna through quantifying the extent to which they co-occur with other freshwater species. We also discuss possible roles for freshwater megafauna as flagship species. Third, we suggest priority scientific and policy recommendations to foster freshwater biodiversity conservation, and we discuss the potential contribution of megafauna conservation to existing multinational environmental agreements. The present results are expected to increase appreciation of freshwater biodiversity and support efforts to halt the largely unnoticed decline of global freshwater biodiversity.

## The status and distribution of freshwater megafauna

We consider all species that require freshwater (or brackish) habitats for completing their entire life cycle as *freshwater species* (He et al. 2017). However, there is no generally accepted definition of freshwater megafauna. Indeed, there is an ongoing debate as to whether body length, mass, trophic level, functional role, human perception and appreciation, or a combination of these characteristics should be applied in defining megafauna (Caro and O’Doherty 1999, Home et al. 2009, Barua et al. 2011, Verissimo et al. 2011). Therefore, we apply a pragmatic definition considering all species with an adult mass of at least 30 kilograms (kg) to be classified as megafauna. A threshold of 30 kg is within the range of those applied to other taxa. For example, in terrestrial systems, a threshold of 15 kg was used for megacarnivores and of 100 kg for megaherbivores (Ripple et al. 2016). In marine systems, a threshold of 44 kg (100 pounds) has been applied (Estes et al. 2016). A commonly used threshold for defining prehistoric megafauna of the Pleistocene is 44 kg (Barnosky 2008).

On the basis of this 30-kg-mass threshold, we compiled a list of freshwater species that meet the threshold and that, because they are well-known or otherwise iconic species, can serve as “ambassadors” representative of both the freshwater megafauna and of conservation priorities for freshwater ecosystems. On this basis, we selected 132 megafauna species, including 73 fishes, 36 reptiles, and 23 mammals (supplemental table S1). We reviewed the global conservation status of these species according to the International Union for Conservation of Nature (IUCN) Red List of Threatened Species^TM^ (hereafter *Red List*; IUCN 2016b). Sixty-two (58%) of the 107 species so far assessed for the Red List are classified as *Threatened*, being *Vulnerable, Endangered*, or *Critically Endangered* (table S1). The baiji (*Lipotes vexillifer)* and the Chinese paddlefish *(Psephurus gladius*) are Critically Endangered (possibly extinct). In addition, six species are *Near Threatened*, six species lack sufficient information to assess their conservation status (*Data Deficient*), and 25 species are *Not Evaluated* for the Red List (table 1). Consequently, the overall level of threat to freshwater megafauna is most likely greater than presented.

**Table 1.** Total number and percentage of the 132 megafauna species classified in each Red List category.

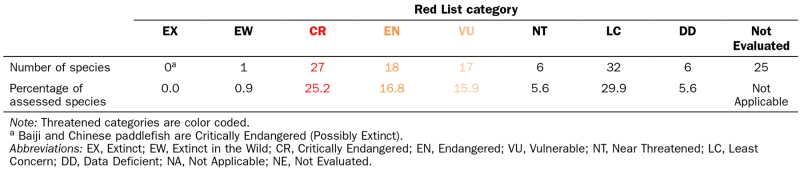

**Figure 2.**
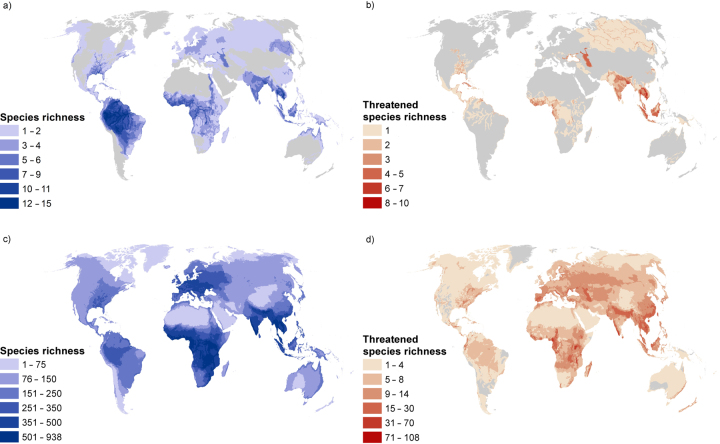
Richness maps: Species richness (a) and threatened species richness (b) of freshwater megafauna. Species richness (c) and threatened species richness (d) of freshwater species exclusive of megafauna (fishes, molluscs, odonates, plants, crabs, crayfish, shrimps, turtles, mammals, birds, and amphibians); gray areas are not inhabited by selected megafauna species. Note that the Americas, Australasia, China, Russia, and parts of the Middle East are incompletely assessed regions; therefore, richness is at least at the level depicted.

Freshwater megafauna inhabit every continent except Antarctica (figure 2a). As we expected, they mostly occur in large rivers (e.g., Amazon, Congo, Ganges, Mekong, and Mississippi) and lakes (e.g., Lake Tanganyika, Tonlé Sap Lake, and the Caspian Sea), which also harbor a major share of the total freshwater fauna (figure 2c). Geographically, South America, Central Africa, and South and Southeast Asia are notably rich in freshwater megafauna. At the same time, South and Southeast Asia contain a relatively high proportion of threatened freshwater megafauna species (figure 2b).

**Figure 3.**
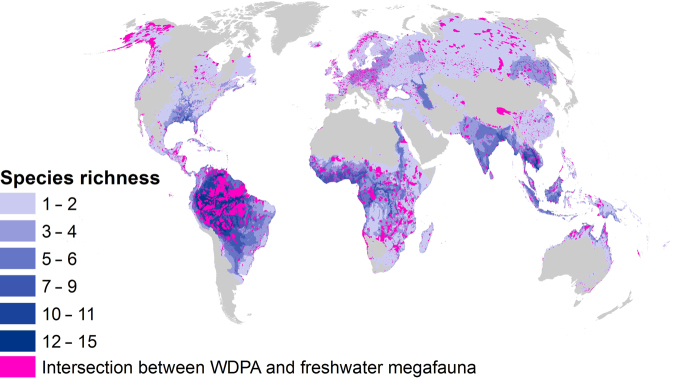
A gap analysis between the World Database on Protected Areas (WDPA) and freshwater megafauna “extant” and “probably extant” records (i.e., PRESENCE scored as 1 or 2 in the Red List) shows that 84% of the collective freshwater megafauna range is outside protected areas; gray areas are not inhabited by selected megafauna species.

Eighty-four percent of the collective freshwater megafauna distribution ranges fall outside of protected areas (figure 3). Only two species, the Baikal seal (*Pusa sibirica*) and Ungava seal (*Phoca vitulina* ssp. *mellonae*), have more than half of their range within protected areas (supplemental table S2). Large rivers show particularly low levels of protected area coverage. For example, the Mekong and Ganges rivers are poorly protected in terms of the proportion of catchment area protected or the maintenance of their natural flow regimes (Abell et al. 2016, Harrison et al. 2016, Abell et al. 2017)—this despite supporting a highly diverse freshwater megafauna. We conclude, therefore, that freshwater species are currently not gaining sufficient conservation attention.

Protected areas are widely considered by the Convention on Biological Diversity (CBD; Leadley et al. 2014) as a primary tool for conservation of biodiversity. The CBD recommends that 17% of terrestrial and freshwater systems should be protected. However, such area-based targets have been shown ineffective in protecting freshwater biodiversity, attributed in part to a current lack of information on the distribution and global extent of wetlands (Watson et al. 2014, Juffe-Bignoli et al. 2016, Abell et al. 2017). Moreover, many protected areas do not incorporate freshwaters as specified conservation targets *per se*; therefore, effective protection is often only incidental and more often absent (Saunders et al. 2002, Pittock et al. 2015, Reis et al. 2017). Rivers, for example, are commonly used to delineate protected area boundaries rather than being considered as a key component of conservation plans (Abell et al. 2007). Where freshwater species, ranges do fall within protected areas, they often remain exposed to threats propagated from outside this area because of pronounced hydrological connectivity gradients up- and downstream (Pittock et al. 2015). However, when thoughtfully selected, megafauna species requirements can guide area targets and boundaries for protected areas, resulting in the major financial support and strong political commitment shown for marine and terrestrial species (Hooker and Gerber 2004, Ripple et al. 2017).

**Figure 4.**
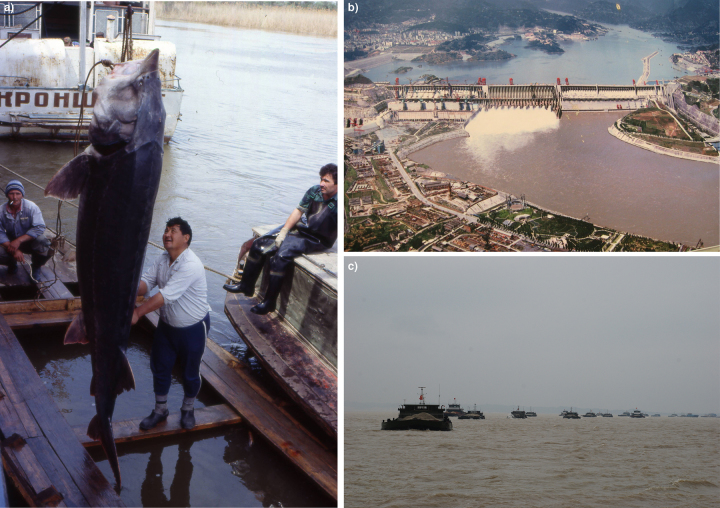
Threats to freshwater megafauna. Left: (a) The beluga (Huso huso) is critically endangered because of overfishing, poaching, and habitat modification. Belugas migrate upstream to spawn; however, impoundments have destroyed most of the species’ spawning grounds. Right top to bottom: (b) The Three Gorges Dam on the Yangtze River increases water temperatures, which causes spawning delays and reduces the spawning activity of the Chinese sturgeon (Acipenser sinensis); (c) boat traffic and pollution, such as from intense sand mining in Poyang Lake (photo from 2010) and associated vessel strikes, are common threats to freshwater megafauna. Photographs: Gerald Zauner, Pedro Vásquez Colmenares, Sonja C. Jähnig; photograph (b) by P. V. Colmenares, published under CC BY-NC 2.0 license (www.flickr.com/photos/pvcg/3412711352/sizes/o).

**Figure 5.**
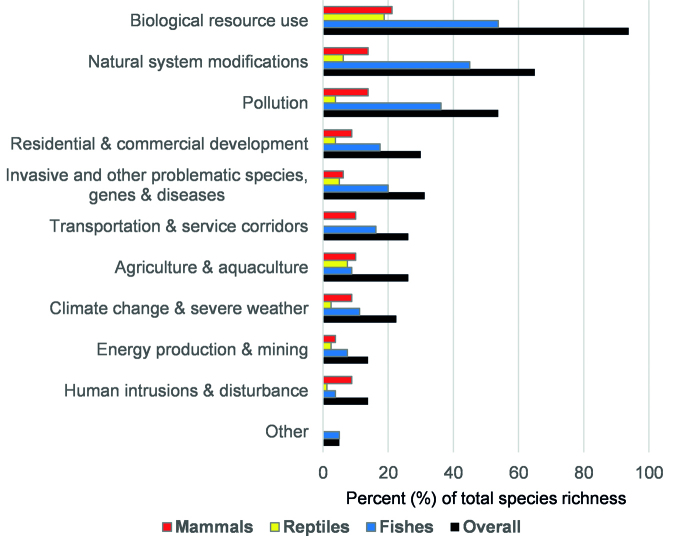
The main threats affecting freshwater megafauna (as a percentage of total species richness).

The key threats to freshwater megafauna species are overexploitation (94% of the analyzed species), habitat alteration (65%), and pollution (54%; figures 4 and 5, and see box 1). The current data suggest that freshwater species are affected by unsustainable population declines caused by humans acting as “superpredators,” as in marine and terrestrial ecosystems (Darimont et al. 2015). In addition to general harvesting for food, “megafishes” are also subject to increasing pressure from anglers as trophy catches (Stone 2007, Maxwell et al. 2016). Water abstraction and dam construction (supplemental tables S3 and S4) alter flow, sediment, and temperature regimes, fragment river networks, and drain and isolate wetlands, thereby affecting home ranges, migratory routes, and access to the spawning sites of megafauna species (Davidson 2014). Agricultural, industrial, and urban pollutants propagate through catchments and affect freshwater megafauna (Pittock et al. 2015). Overall, these threats, single or in combination, lead to a decline of populations, a reduction of genetic variability, and ultimately to species extinction (He et al. 2017).

Box 1. **Charismatic freshwater megafauna species.**

Freshwater megafauna species require freshwater (or brackish) habitat for completing any critical stage in the species life cycle. In addition to their potential to act as flagship species, megafauna species fulfill important ecological roles, such as ecosystem engineers: For example, the large hippo (*Hippopotamus amphibius*; figure 1) alters floodplain habitats and the river morphology and fertilizes floodwaters, which has an effect on the productivity of fish populations. The large hippo is primarily threatened by illegal hunting and loss of habitats due to conflicting human population growth, development, and agriculture. The Nile crocodile (*Crocodylus niloticus*; figure 1) was hunted for its skin almost to extinction in many locations but was rather successfully protected because of the development of crocodile farming, which now satisfies human demands. After years of being classified as *Endangered*, in 1996, the status of the Nile crocodile improved to *Least Concern* (but the Red List notes this status requires updating). The beluga, or European sturgeon (*Huso huso*; figures 1 and 4), is the largest freshwater fish in the world, as is demonstrated by the life-size model at the National Park Donau-Auen offices in Austria.

Because most megafauna species are threatened by multiple pressures, an integrated management approach is required to protect and increase their populations over the long term (Abell et al. 2007, Pittock et al. 2015). An immediate priority is to address overexploitation. However, protected areas alone will not be sufficient to protect and improve freshwater megafauna, certainly while harvesting remains unsustainable. The impacts of the global boom on hydropower dams, such as in the Amazon, Congo, and Mekong river basins (Zarfl et al. 2015, Winemiller et al. 2016), also represent priority areas for attention if freshwater species declines are to be reversed. Unsustainable abstraction of water is likewise a major concern in the dry regions of the world, such as the Ganges-Brahmaputra and Indus river basins.

## Co-occurrence of freshwater megafauna with other freshwater biodiversity

According to the most comprehensive, spatially explicit biodiversity data set available up to now, 93% of all assessed freshwater biodiversity co-occurs with the freshwater megafauna species (figure 2, supplemental table S5.1). Overall, 60% of the world's threatened freshwater species are found within the collective freshwater megafauna range, varying from 24% (odonates) to 87% (turtles; table S5.2). Indeed, the level of co-occurrence is expected to be even higher because the spatial distribution and the conservation status of freshwater biodiversity are not yet fully assessed for many regions of the world. Therefore, effective conservation of megafauna species will most likely benefit many additional freshwater species. A similar umbrella effect has recently been demonstrated for terrestrial megafauna (Branton and Richardson 2011, Ripple et al. 2016). For example, conservation efforts targeting the giant panda (*Ailuropoda melanoleuca*) in China (Li and Pimm 2016) protect co-occurring species such as the threatened golden snub-nosed monkey (*Rhinopithecus roxellana)*, blackthroat (*Calliope obscura*), and Liangbei toothed toad (*Oreolalax liangbeiensis*). Similarly, the jaguar conservation network in South America, established to maintain habitat quality and connectivity, benefits co-occurring mammal species such as the threatened lowland tapir (*Tapirus terrestris*; Thornton et al. 2016).

Whether such an umbrella effect can be realized strongly depends on the role megafauna species have on ecosystem functioning (Ford et al. 2017). Although freshwater megafauna might take a central role in food webs (Brose et al. 2016), for most species, their ecological role is yet to be determined. The presence of top-down or bottom-up processes is likely to determine the potential wider benefits of their conservation such that, in some cases, smaller species might be more effective as conservation priorities (Ford et al. 2017). However, it has been argued that top-down control is greater in water than on land (Shurin et al. 2002 and references therein). At the same time, we need to be aware that it may be challenging to develop effective conservation strategies for freshwater megafauna species on account of their large home ranges, complex life cycles, and distinct movement dynamics.

In addition, conservation efforts for freshwater biodiversity must consider headwater rivers and streams. Although headwaters themselves contain few megafauna species, they are essential in supporting the biodiversity of entire river systems, including megafauna species present in downstream sections (Meyer et al. 2007).

## Knowledge gaps and next steps

Information gaps on the global distribution and status of freshwater megafauna need to be filled to ensure evidence-based and effective conservation strategies, regionally and globally. One priority is to identify sites of importance to conservation of freshwater species. Key Biodiversity Areas (KBAs), defined as “sites contributing significantly to the global persistence of biodiversity” (IUCN 2016a), need to be identified and validated for freshwaters for most of the world (but see Holland et al. 2012).

Evidence-based conservation planning depends on baseline information on species; this includes regularly updated and comprehensive Red List assessments, with a priority focus on additional research for Data Deficient species and new assessments of the many species yet to be evaluated. Conservation planning might also focus on the identification of evolutionarily distinct and globally endangered (EDGE) species (ZSL 2016) based on an updated phylogeny. Such baseline information would include refined and validated distribution maps, including spawning areas and migration routes. Eventually, a freshwater megafauna Red List index could be developed to track change over time within global monitoring programs.

On the basis of the information for critical sites and species, systematic conservation planning approaches, as opposed to *ad hoc* conservation planning (Hermoso et al. 2015), may further help improve the representation of freshwater biodiversity within protected area networks. However, climate change impacts on megafauna distributions have to be considered, too, in particular in relation to potential boundary modifications for protected areas (Pittock et al. 2015).

Finally, long-term data are available for only a few mainly commercially important megafauna populations, such as Chinook salmon (*Oncorhynchus tshawytscha*), Atlantic salmon (*Salmo salar*), sturgeons, or crocodiles. Such data are fundamental to tracking the status and the trends of megafauna species (WWF 2016).

The potential conservation benefits of flagship and umbrella freshwater species, sometimes referred to as “freshwater pandas” (Kalinkat et al. 2017), have only been considered for a few regions. Ebner and colleagues (2016), for example, presented Australian freshwater flagship species, including several megafauna species, arguing for an audience-targeted nomination of species that would receive conservation action. Promotion of flagship species needs to be targeted to specific regions and/or stakeholders, such as recreational or commercial ­fishers, scientists, ­environmental managers, water-resource users, or indigenous ­people, to consider their differing perceptions of nature and ­biodiversity (Cooke et al. 2013). Successful examples for such targeted flagship promotion are the largetooth sawfish (*Pristis pristis*) or the smaller-bodied axolotl (*Ambystoma mexicanum*; Bride et al. 2008, Barua et al. 2011, Ebner et al. 2016). Likewise, the identification of threats common to all species (Donaldson et al. 2016) is an essential precursor to the development of ­effective ­management strategies benefiting both ­megafauna and other co-occurring species.

The contribution of freshwater megafauna to the provision of ecosystem services requires further investigation. Many species are of importance to livelihoods, such as through contributions to national and local fisheries (Petrere et al. 2004), recreational fisheries (Jensen et al. 2009), or tourism (Solomon et al. 2004). Freshwater megafauna such as the taimen (Hucho taimen) and other large fishes are already known to be important for recreational fisheries (Granek et al. 2008), and other species, such as river dolphins, bring important tourism benefits (de Sá Alves et al. 2012).

Closing the knowledge gaps for freshwater megafauna will help achieve two major goals: (1) raising political will as needed to conserve freshwater megafauna and freshwater biodiversity in general and (2) identifying flagship species targeted to specific regions or stakeholders (Verissimo et al. 2011).

## Policy relevance

To counteract the ongoing decline in freshwater biodiversity, conservation actions are required at multiple spatial scales (Sodhi et al. 2011). At the local scale, priority ­activities include habitat restoration, the creation of protected freshwater areas, the control of illegal hunting, and recovery plans for threatened species. At the regional scale, measures include cooperation among neighboring countries, such as regulation of international wildlife trade and transboundary river-basin management. At the global scale, the impact of climate change on freshwater ecosystems has to be addressed.

Multinational environmental agreements aim to improve the status of freshwater biodiversity, such as through regulating trade and advocating international cooperation. For example, 29 freshwater megafauna species are represented in the Convention on Migratory Species (CMS), and 74 species are listed in the Convention on International Trade in Endangered Species of Wild Fauna and Flora (CITES; supplemental table S6). The Secretariat of the CMS has already recognized the need to strengthen measures to protect transboundary migratory freshwater fishes, which include many of the megafishes (Stone 2007, Hogan 2011). Improved knowledge of freshwater megafauna and leveraging megafauna species to generate attention and action for freshwater biodiversity could help achieve the targets of international conventions.

For example, actions implemented for conservation of freshwater megafauna could simultaneously help reach multiple Aichi Targets: reducing the fragmentation and degradation of freshwater habitats (target 5), improving the long-term sustainability of freshwater fisheries (target 6), decreasing pollution effects in freshwater ecosystems (target 8), improving the effectiveness of freshwater protected areas (target 11), and closing data gaps regarding the conservation status of freshwater species, which will allow better monitoring of trends in species extinctions and the implementation of actions to reverse those trends (target 12). Moreover, knowledge of and attention to freshwater megafauna can support the Ramsar Convention to maintain or restore the ecological character of Ramsar sites through effective planning and integrated management (target 5 of Ramsar's 2016–2024 Strategic Plan; Resolution XII.2; Ramsar 2015). Freshwater megafauna can also highlight and help shape the application of two targets of the Sustainable Development Goals (SDGs): “The protection and restoration of water-related ecosystems, including wetlands, rivers, aquifers and lakes” (target 6.6) and “the conservation, restoration, and sustainable use of terrestrial and inland freshwater ecosystems and their services” (target 15.1; UN 2016). Associated with target 15 is the process of safeguarding terrestrial and freshwater key biodiversity areas around the world. Finally, megafauna data can be used to identify transboundary basins where large migratory fishes provide important natural resources that benefit multiple nations and guide management decisions for programs such as the Intergovernmental Platform on Biodiversity and Ecosystem Services (Díaz et al. 2015), the Transboundary Waters Assessment Programme (TWAP), and the UN Watercourses Convention 2015 (Verissimo et al. 2011, UNWC 2016).

## Conclusions

Freshwater is both a resource for human use as well as part of a diverse mix of ecosystems containing a unique biodiversity. The unusually large and fast decline in freshwaters is a product of their relatively small extent and distinct internal connectivity, as well as with their close links to surrounding terrestrial areas. Freshwater ecosystems are quickly and significantly affected by the overharvesting of regional fishes, shellfishes, and plants; the overabstraction of water; pollution; and the fragmentation of rivers. The effective management of these threats is further complicated when river catchments cross political or administrative borders (WWF 2016).

Despite these major challenges and the high value of freshwater ecosystems in terms of biodiversity, livelihoods, and economics, the fact that freshwater ecosystems are declining at greater rates than other systems suggests that there is less investment in their conservation and management. Therefore, there is a major conflict between the human use of freshwater and the conservation of freshwater ecosystems. Given that the availability of freshwater, both spatially and temporally, is predicted to decrease in many regions in the future, this conflict is likely to increase. Solutions to the water-supply crisis have focused on engineering approaches, such as the construction of dams for water storage and power generation, interbasin water transfers, or the construction of dikes and channels for flood protection. Frequently, these measures will accelerate the decline in freshwater biodiversity as fundamental habitats and connectivity are degraded or lost (Vörösmarty et al. 2010, Green et al. 2015, Harrison et al. 2016). However, resolving this conflict may be possible if the ecosystem services provided by diverse and intact freshwaters become more widely acknowledged and the ­species in freshwaters become better known and valued.

Freshwater megafauna have a great potential—yet to unfold—to communicate to the public, to policymakers, and to donors the immense value of freshwater ecosystems, including a unique biodiversity. Here, we provide spatially explicit and quantitative data supporting a better use of freshwater megafauna as a conservation tool. Our results and recommendations demonstrate the potential for freshwater megafauna to generate greater public awareness and political will to better support the conservation of freshwater ecosystems and to stop—or even reverse—their current widespread and tragic decline.

## Supplementary Material

Supplemental data Supplementary data are available at *BIOSCI* online.
