# Supplementary material for: Freshwater Megafauna: Flagships for Freshwater Biodiversity under Threat
Source: Bioscience. 2017 Sep 20;67(10):919–27. doi: 10.1093/biosci/bix099 (PMC5862257; doi:10.1093/biosci/bix099)
Supplement: Supplemental data — Supplementary data are available at BIOSCI online. [file bix099_supp.docx]

Supplemental material (Carrizo et al. – Megafauna, doi:10.1093/biosci/bix099)

Material and Methods

Data

The IUCN Red List of Threatened Species^TM^

The Red List assessment process assigns a species to one of the following Categories as a measure of the risk of global extinction: Least Concern (LC), Near Threatened (NT), Vulnerable (VU), Endangered (EN), Critically Endangered (CR), Extinct in the Wild (EW), Extinct (EX) and Data Deficient (DD) (IUCN 2016). The data exported from the Red List still included some Categories that have been decommissioned (i.e., no longer used). These Categories were combined with the appropriate current Red List Categories as follows; species assessed as Lower Risk/conservation dependent (LR/cd) and Lower Risk/near threatened (LR/nt) were incorporated within the current Category NT, and species assessed as Lower Risk/least concern (LR/lc) were incorporated within the Category LC. The decommissioned Categories will gradually be removed from the Red List as the species within them are reassessed according to the current Categories. Along with assigning a Red List Category, the Red List assessments collate information including threats, habitats, country occurrence, and use and trade. Our Red List export was current as of 5th May 2016.

Geographic distributions

Where possible, species geographic distributions are mapped as part of the Red List assessment process. The distributions are mapped to sub-catchments allowing delineation and analysis, and hence conservation planning and practical management interventions, to take place at the appropriate ecological scale. Mapping to sub-catchments also enables consideration of biological and ecological processes, such as species migrations and threat propagation, mediated by catchment connectivity (Nel et al. 2009). To define sub-catchments, we used HydroBASINS, a global standardized hydrological framework that delineates catchments at multiple resolutions and includes network connectivity information (Lehner B. 2012, Lehner Bernhard and Grill 2013). We analyzed distribution data at the HydroBASINS level 8 sub-catchment scale (catchment area: 538.3 ± 649.45 km^2^; mean ± SD). ‘Presence’ within each sub-catchment is indicated according to the nature and certainty of occurrence, based on observation and inference, and is coded as Extant, Probably Extant, Possibly Extant, Possibly Extinct, Extinct (post 1500), and Presence Uncertain. ‘Origin’ is coded as Native, Reintroduced, Introduced, Vagrant, and Origin Uncertain (www.iucnredlist.org/technical-documents/spatial-data). We spatially transferred existing reptile, mammal, and fish distribution maps that were not originally mapped to HydroBASINS sub-catchments via a semi-automated process. Finally, we individually validated these maps to ensure correct data transfer to the catchment framework. Where a map was not available, we created a map based on descriptions from the IUCN Red List and other sources.

For the overall freshwater biodiversity assessment, we used species distribution data for eleven freshwater groups: 1776 plants, 1505 odonates, 1277 crabs, 4221 amphibians, 2182 birds, 141 mammals, 734 shrimps, 504 crayfish, 270 turtles, 2021 molluscs, and 6647 fishes. Not all of these groups are comprehensively assessed on the Red List, but we included all available data to also inform conservation of those partially assessed groups.

World Database on Protected Areas (WDPA)

For the gap analysis, we performed spatial coverage analyses using the World Database on Protected Areas (WDPA) dataset (IUCN and UNEP-WCMC 2016). We used a pre-processed layer provided by the United Nations Environment Programme World Conservation Monitoring Centre (UNEP WCMC). In this layer, all points and polygons with STATUS = “not reported” and STATUS = “proposed” and all UNESCO MAB (The United Nations Educational, Scientific and Cultural Organization Man and the Biosphere Programme) reserves had been removed. These data were removed because the features may include large areas that do not meet the definition of protected areas (Juffe-Bignoli et al. 2014). Our WDPA layer was current as of April 2016.

CMS and CITES

We sourced information on species representation in the Convention on Migratory Species (CMS) and the Convention on International Trade in Endangered Species of Wild Fauna and Flora (CITES) from the Species+ database (www.speciesplus.net/). The CMS Appendix I lists threatened migratory species and CMS Appendix II lists migratory species requiring international cooperation ([www.cms.int/en](http://www.cms.int/en)). CITES Appendix I lists “species that are the most endangered among CITES-listed animals and plants. They are threatened with extinction and CITES prohibits international trade in specimens of these species except when the purpose of the import is not commercial...”. CITES Appendix II lists “species that are not necessarily now threatened with extinction but that may become so unless trade is closely controlled.” CITES Appendix III lists “species included at the request of a Party that already regulates trade in the species and that needs the cooperation of other countries to prevent unsustainable or illegal exploitation” (www.cites.org)..

Analysis

Species list

We selected 132 freshwater megafauna species (table S1) with reference to published weight data (table S7).

Conservation status

We collated IUCN Red List assessment data for all 132 species, where available, as of May 5th 2016. We summarized conservation status of the freshwater megafauna as the mid-point estimate (MID) of the percentage of threatened species (i.e., assuming the Data Deficient (DD) species are threatened in the same proportion as the species for which there are sufficient data) as follows: % threat = (CR + EN + VU) / (total assessed - EX - EW - DD).

Geographic distributions

We derived global species richness maps from the individual species Red List maps. We summarized the spatial data into species richness and threatened species richness maps for megafauna and overall freshwater biodiversity, using ‘Extant’ (PRESENCE = 1) and ‘Probably Extant’ (PRESENCE = 2) records within each HydroBASINS sub-catchment (figure 2). Furthermore, we used the distribution data on the remaining freshwater species to run a co-occurrence analysis with the megafauna (tables S5.1 and 5.2).

Threats

We extracted threat data, collated during Red List assessments from the IUCN Species Information Service (SIS) for each species where available. We summarized the percentage of species affected by each threat category for mammals, fishes, reptiles, and megafauna overall. We reviewed the threats at each of the three levels (levels 0, 1, and 2) of detail within the threat classification hierarchy (figure 5 and tables S3 & S4).

Gap analysis

We filtered the species ranges to only include ‘Extant’ (PRESENCE = 1) and ‘Probably Extant’ (PRESENCE = 2) records. We projected all spatial data to WGS_1984 Cylindrical Equal Area to ensure correct calculation of areas. We then intersected the set of corresponding HydroBASINS with the WDPA layer. We aggregated the total area (km^2^) of each HydroBASINS that is overlapped by a protected area from the individual intersecting segments. Thus, we calculated the area of each species range that is covered by protected areas from the component HydroBASINS overlaps (table S2). We visualized the WDPA overlaps with the ‘Extant’ and ‘Probably Extant’ records of megafauna to evaluate the spatial distribution of gaps (figure 3).

CMS and CITES

We summarized the presence of species within the CMS and CITES conventions (table S6).

All analyses were conducted using custom R scripts with R version 2.15.2 (R Core Team 2012) and ArcGIS (ESRI 2015).

References (supplemental material)

ESRI. 2015. ArcGIS Desktop: Release 10.3.1. Redlands, CA: Environmental Systems Research

IUCN. 2016. IUCN Red List of Threatened Species. Version 2016.1. (5 May 2016; www.iucnredlist.org)

IUCN and UNEP-WCMC. 2016. The World Database on Protected Areas (WDPA). (10 April 2016; www.protectedplanet.net)

Juffe-Bignoli D, et al. 2014. Protected Planet Report 2014. UNEP-WCMC.

Lehner B. 2012. HydroBASINS Version1.b. Global watershed boundaries and sub-basin delineation derived from HydroSHEDS data at 15 second resolution. (20 January 2016; www.hydrosheds.org).

Lehner B, Grill G. 2013. Global river hydrography and network routing: baseline data and new approaches to study the world’s large river systems. Hydrological Processes 27:2171-2186.

Nel JL, Roux DJ, Abell R, Ashton PJ, Cowling RM, Higgins JV, Thieme M, Viers JH. 2009. Progress and challenges in freshwater conservation planning. Aquatic Conservation: Marine and Freshwater Ecosystems 19:474-485.

R Core Team. 2012. R: a language and environment for statistical computing. R Foundation for Statistical Computing, Vienna, Austria.

Table S1.

Species list and status from the International Union for Conservation of Nature (IUCN) Red List of Threatened species^TM^. Sorted by Red List Category EX, Extinct; EW, Extinct in the Wild; CR, Critically Endangered; EN, Endangered; VU, Vulnerable; NT, Near Threatened; LC, Least Concern; DD, Data Deficient; NE, Not Evaluated.

| **Binomial** | **Common Name** | **Class** | **Red List Category** |
| --- | --- | --- | --- |
| *Nilssonia nigricans* | Black Soft-shell Turtle, Black Softshell Turtle | Reptilia | EW |
|  |  |  |  |
| *Acipenser gueldenstaedtii* | Russian sturgeon | Actinopterygii | CR |
| *Acipenser mikadoi* | Sakhalin Sturgeon | Actinopterygii | CR |
| *Acipenser nudiventris* | Ship Sturgeon, Spiny Sturgeon | Actinopterygii | CR |
| *Acipenser persicus* | Persian Sturgeon | Actinopterygii | CR |
| *Acipenser schrenckii* | Amur Sturgeon | Actinopterygii | CR |
| *Acipenser sinensis* | Chinese Sturgeon | Actinopterygii | CR |
| *Acipenser stellatus* | Stellate Sturgeon, Sevruga, Star Sturgeon | Actinopterygii | CR |
| *Acipenser sturio* | Common Sturgeon, Atlantic Sturgeon, Baltic Sturgeon, German Sturgeon | Actinopterygii | CR |
| *Catlocarpio siamensis* | Giant Carp, Giant Barb | Actinopterygii | CR |
| *Huso dauricus* | Kaluga | Actinopterygii | CR |
| *Huso huso* | Beluga, Giant Sturgeon, European Sturgeon, Great Sturgeon | Actinopterygii | CR |
| *Maccullochella peelii* | Murray Cod, Murray River Cod | Actinopterygii | CR |
| *Pangasianodon gigas* | Mekong Giant Catfish, Giant Catfish | Actinopterygii | CR |
| *Pangasius sanitwongsei* | Giant Pangasius, Paroon Shark, Pangasid-catfish, Pla Thepa | Actinopterygii | CR |
| *Pristis pristis* | Largetooth Sawfish | Chondrichthyes | CR |
| *Pristis pectinata* | Smalltooth Sawfish, Wide Sawfish | Chondrichthyes | CR |
| *Psephurus gladius* | Chinese Paddlefish | Actinopterygii | CR* |
| *Lipotes vexillifer* | Yangtze River Dolphin, Whitefin Dolphin, White Flag Dolphin, Chinese Lake Dolphin, Changjiang Dolphin | Mammalia | CR* |
| *Neophocaena asiaeorientalis* ssp. *asiaeorientalis* | Yangtze Finless Porpoise | Mammalia | CR |
| *Alligator sinensis* | Chinese Alligator, China Alligator | Reptilia | CR |
| *Chitra chitra* | Southeast Asian Narrow-headed Softshell Turtle, Striped Narrow-headed Softshell Turtle | Reptilia | CR |
| *Crocodylus intermedius* | Orinoco Crocodile | Reptilia | CR |
| *Crocodylus rhombifer* | Cuban Crocodile | Reptilia | CR |
| *Crocodylus siamensis* | Siamese Crocodile | Reptilia | CR |
| *Gavialis gangeticus* | Gharial, Indian Gharial, Fish-eating Crocodile, Indian Gavial, Gavial, Long-nosed Crocodile | Reptilia | CR |
| *Mecistops cataphractus* | Slender-snouted Crocodile, African Slender-snouted Crocodile | Reptilia | CR |
| *Rafetus swinhoei* | Yangtze Giant Softshell Turtle | Reptilia | CR |
|  |  |  |  |
| *Acipenser baerii* | Siberian sturgeon | Actinopterygii | EN |
| *Argyrosomus hololepidotus* | Madagascar Kob, Madagascar Meagre | Actinopterygii | EN |
| *Himantura polylepis* | Giant freshwater stingray | Chondrichthyes | EN |
| *Hucho hucho* | Danube Salmon, Huchen | Actinopterygii | EN |
| *Hypselobarbus mussullah* | Hump Backed Mahseer | Actinopterygii | EN |
| *Lates angustifrons* | Tanganyika Lates | Actinopterygii | EN |
| *Probarbus jullieni* | Jullien's Golden Carp, Seven-striped Barb | Actinopterygii | EN |
| *Probarbus labeamajor* | Thicklipped Barb | Actinopterygii | EN |
| *Scaphirhynchus albus* | Pallid Sturgeon | Actinopterygii | EN |
| *Tor putitora* | Putitor Mahseer, Golden Mahaseer | Actinopterygii | EN |
| *Choeropsis liberiensis* | Pygmy Hippopotamus | Mammalia | EN |
| *Kobus megaceros* | Nile Lechwe | Mammalia | EN |
| *Platanista gangetica* ssp. *gangetica* | Ganges River Dolphin, Ganges Susu, Ganges Dolphin | Mammalia | EN |
| *Platanista gangetica* ssp. *minor* | Indus River Dolphin, Susu, Indus Dolphin | Mammalia | EN |
| *Pusa caspica* | Caspian Seal | Mammalia | EN |
| *Chitra indica* | Indian Narrow-headed Softshell Turtle, Narrow-headed Softshell Turtle | Reptilia | EN |
| *Orlitia borneensis* | Bornean River Turtle, Malaysian Giant Turtle | Reptilia | EN |
| *Pelochelys cantorii* | Cantor's Giant Softshell Turtle, Frog-faced Softshell Turtle | Reptilia | EN |
|  |  |  |  |
| *Hucho taimen* | Siberian Taimen, Mongolian Taimen, Siberian Salmon, Taimen | Actinopterygii | VU |
| *Luciobarbus esocinus* | Pike Barbel | Actinopterygii | VU |
| *Megalops atlanticus* | Tarpon | Actinopterygii | VU |
| *Polyodon spathula* | Paddlefish, Spadefish, Duckbill Cat, Spoonbill Cat | Actinopterygii | VU |
| *Hippopotamus amphibius* | Hippopotamus, Large Hippo, Common Hippopotamus | Mammalia | VU |
| *Orcaella brevirostris* | Irrawaddy Dolphin, Snubfin Dolphin | Mammalia | VU |
| *Trichechus inunguis* | Amazonian Manatee, South American Manatee | Mammalia | VU |
| *Trichechus manatus* | American Manatee, West Indian Manatee | Mammalia | VU |
| *Trichechus senegalensis* | African Manatee, Seacow, West African Manatee | Mammalia | VU |
| *Amyda cartilaginea* | Asiatic Softshell Turtle, Southeast Asian Softshell Turtle | Reptilia | VU |
| *Crocodylus acutus* | American Crocodile | Reptilia | VU |
| *Crocodylus palustris* | Mugger, Muggar, Broad-snouted Crocodile, Marsh Crocodile | Reptilia | VU |
| *Macrochelys temminckii* | Alligator Snapping Turtle | Reptilia | VU |
| *Nilssonia leithii* | Leith's Softshell Turtle | Reptilia | VU |
| *Osteolaemus tetraspis* | African Dwarf Crocodile, West African Dwarf Crocodile | Reptilia | VU |
| *Pelochelys bibroni* | Asian Giant Softshell Turtle, Striped New Guinea Softshell Turtle | Reptilia | VU |
| *Tomistoma schlegelii* | False Gharial, Tomistoma, Sunda Gharial, Malayan Gharial | Reptilia | VU |
|  |  |  |  |
| *Acipenser medirostris* | Green Sturgeon | Actinopterygii | NT |
| *Acipenser oxyrinchus* | Gulf Sturgeon | Actinopterygii | NT |
| *Arius gigas* | Giant Sea Catfish | Actinopterygii | NT |
| *Carcharhinus leucas* | Bull Shark | Chondrichthyes | NT |
| *Hypophthalmichthys molitrix* | Silver Carp | Actinopterygii | NT |
| *Wallago attu* | Wallago (Giant sheatfish) | Actinopterygii | NT |
|  |  |  |  |
| *Acipenser fulvescens* | Lake Sturgeon | Actinopterygii | LC |
| *Acipenser transmontanus* | White Sturgeon | Actinopterygii | LC |
| *Chrysichthys cranchii* | Kokuni, Kokuni, Manora | Actinopterygii | LC |
| *Clarias gariepinus* | African Catfish, Sharptooth Catfish, Catfish, Common Catfish, Mudfish, Barbel, Sharptoothed Catfish | Actinopterygii | LC |
| *Hemibagrus maydelli* | Krishna Mystus | Actinopterygii | LC |
| *Hemibagrus wyckioides* | Asian Red Tailed Catfish, Red fin bagrus | Actinopterygii | LC |
| *Heterobranchus longifilis* | Catfish, Sampa, Vundu, Vundu | Actinopterygii | LC |
| *Hydrocynus goliath* | Giant tigerfish, Giant tigerfish | Actinopterygii | LC |
| *Ictalurus furcatus* | Blue Catfish | Actinopterygii | LC |
| *Labeo rohita* | Rohu | Actinopterygii | LC |
| *Lates niloticus* | Nile Perch, Victoria Perch, African Snook | Actinopterygii | LC |
| *Morone saxatilis* | Striped Bass | Actinopterygii | LC |
| *Pylodictis olivaris* | Flathead Catfish | Actinopterygii | LC |
| *Salmo salar* | Atlantic Salmon, Black Salmon | Actinopterygii | LC |
| *Salmo trutta* | Brown Trout, Sea Trout | Actinopterygii | LC |
| *Silurus glanis* | Wels Catfish | Actinopterygii | LC |
| *Hydrochoerus hydrochaeris* | Capybara | Mammalia | LC |
| *Kobus leche* | Southern Lechwe | Mammalia | LC |
| *Pusa sibirica* | Baikal Seal | Mammalia | LC |
| *Tragelaphus spekii* | Sitatunga, Marshbuck | Mammalia | LC |
| *Alligator mississippiensis* | American Alligator, Mississippi Alligator | Reptilia | LC |
| *Apalone ferox* | Florida Softshell Turtle | Reptilia | LC |
| *Caiman crocodilus* | Common Caiman, Spectacled Caiman | Reptilia | LC |
| *Caiman latirostris* | Broad-snouted Caiman | Reptilia | LC |
| *Caiman yacare* | Yacaré | Reptilia | LC |
| *Crocodylus johnsoni* | Australian Freshwater Crocodile, Johnson's Crocodile, Freshie, Johnstone's Crocodile, Johnston's Crocodile | Reptilia | LC |
| *Crocodylus moreletii* | Morelet's Crocodile, Belize Crocodile | Reptilia | LC |
| *Crocodylus niloticus* | Nile Crocodile | Reptilia | LC |
| *Crocodylus porosus* | Salt-Water Crocodile, Estuarine Crocodile | Reptilia | LC |
| *Melanosuchus niger* | Black Caiman | Reptilia | LC |
| *Paleosuchus palpebrosus* | Dwarf Caiman, Cuvier's Smooth-fronted Caiman | Reptilia | LC |
| *Podocnemis expansa* | South American River Turtle, Arrau, Giant South American Turtle, Tartaruga | Reptilia | LC |
|  |  |  |  |
| *Arapaima gigas* | Arapaima, Pirarucu | Actinopterygii | DD^a^ |
| *Potamotrygon brachyura* | Giant Freshwater Stingray | Chondrichthyes | DD |
| *Scomberomorus sinensis* | Chinese Seerfish | Actinopterygii | DD |
| *Wallago micropogon* | Walaga | Actinopterygii | DD |
| *Inia geoffrensis* | Amazon River Dolphin, Boutu | Mammalia | DD |
| *Sotalia fluviatilis* | Tucuxi, Bouto Dolphin | Mammalia | DD |
|  |  |  |  |
| *Arapaima agassizii* | n/a | Actinopterygii | NE^a^ |
| *Arapaima leptosoma* | n/a | Actinopterygii | NE^a^ |
| *Arapaima mapae* | n/a | Actinopterygii | NE^a^ |
| *Atractosteus spatula* | Alligator gar | Actinopterygii | NE |
| *Brachyplatystoma filamentosum* | Kumakuma | Actinopterygii | NE |
| *Colossoma macropomum* | Cachama | Actinopterygii | NE |
| *Ctenopharyngodon idella* | Grass Carp | Actinopterygii | NE |
| *Eleutheronema tetradactylum* | Fourfinger threadfin | Actinopterygii | NE |
| *Lates calcarifer* | Barramundi | Actinopterygii | NE |
| *Oncorhynchus tshawytscha* | Chinook Salmon | Actinopterygii | NE |
| *Polydactylus macrochir* | Grand Threadfin | Actinopterygii | NE |
| *Pseudoplatystoma corruscans* | Spotted sorubim | Actinopterygii | NE |
| *Pseudoplatystoma fasciatum* | Barred sorubim | Actinopterygii | NE |
| *Silurus soldatovi* | Soldatov's catfish | Actinopterygii | NE |
| *Wallago leerii* | Tapah | Actinopterygii | NE |
| *Zungaro zungaro* | Guilded Catfish | Actinopterygii | NE |
| *Inia araguaiaensis* | Araguainan River dolphin | Mammalia | NE |
| *Inia boliviensis* | Bolivian River dolphin | Mammalia | NE |
| *Phoca vitulina* ssp. *mellonae* | Seal Lake Seal or Ungava Seal | Mammalia | NE |
| *Pusa hispida* ssp. *ladogensis* | Ladoga Seal | Mammalia | NE |
| *Pusa hispida* ssp. *saimensis* | Saimaa Ringed Seal | Mammalia | NE |
| *Chitra vandijki* | Burmese Narrow-Headed Softshell Turtle | Reptilia | NE |
| *Eunectes murinus* | Anaconda | Reptilia | NE |
| *Osteolaemus osborni* | Osborn's dwarf crocodile | Reptilia | NE |
| *Trionyx triunguis* | African Softshell Turtle | Reptilia | NE |

*Note:* ^a^Covered by *Arapaima* spp. complex map. *Possibly Extinct.

Table S2.

Protected area (PA) coverage (km^2^) per species for catchments where the species is tagged as ‘Extant’ or ‘Probably Extant’ (PRESENCE = 1 and 2).

| **Binomial** | **Range Area (km^2^)** | **PA Coverage (km^2^)** | **% Coverage** |
| --- | --- | --- | --- |
| *Pusa sibirica* | 32835 | 32718 | 99.6 |
| *Phoca vitulina* ssp. *mellonae* | 8257 | 7120 | 86.2 |
| *Kobus leche* | 334770 | 163472 | 48.8 |
| *Colossoma macropomum* | 6821773 | 2945334 | 43.2 |
| *Zungaro zungaro* | 6825621 | 2945587 | 43.2 |
| *Brachyplatystoma filamentosum* | 7387788 | 3130930 | 42.4 |
| *Melanosuchus niger* | 6121171 | 2572893 | 42.0 |
| *Podocnemis expansa* | 6012060 | 2520214 | 41.9 |
| *Inia geoffrensis* | 2451980 | 1025499 | 41.8 |
| *Trichechus inunguis* | 1548182 | 645008 | 41.7 |
| *Arapaima* spp. | 3020991 | 1257287 | 41.6 |
| *Inia boliviensis* | 162469 | 66876 | 41.2 |
| *Sotalia fluviatilis* | 1318983 | 538836 | 40.9 |
| *Crocodylus intermedius* | 619088 | 237930 | 38.4 |
| *Caiman crocodilus* | 9510272 | 3386756 | 35.6 |
| *Pseudoplatystoma fasciatum* | 10027741 | 3370646 | 33.6 |
| *Paleosuchus palpebrosus* | 10600204 | 3476516 | 32.8 |
| *Pusa hispida* ssp. *saimensis* | 5512 | 1773 | 32.2 |
| *Oncorhynchus tshawytscha* | 2615911 | 838485 | 32.1 |
| *Hippopotamus amphibius* | 3105015 | 958757 | 30.9 |
| *Megalops atlanticus* | 1053043 | 313900 | 29.8 |
| *Hydrochoerus hydrochaeris* | 12609846 | 3730146 | 29.6 |
| *Lates calcarifer* | 196605 | 57373 | 29.2 |
| *Eunectes murinus* | 5571884 | 1524645 | 27.4 |
| *Hucho hucho* | 141200 | 35611 | 25.2 |
| *Tor putitora* | 48328 | 11927 | 24.7 |
| *Kobus megaceros* | 177684 | 40584 | 22.8 |
| *Trichechus manatus* | 225075 | 51217 | 22.8 |
| *Pristis pectinata* | 174098 | 39109 | 22.5 |
| *Choeropsis liberiensis* | 166731 | 37398 | 22.4 |
| *Hydrocynus goliath* | 476718 | 100609 | 21.1 |
| *Tragelaphus spekii* | 4710209 | 976576 | 20.7 |
| *Polydactylus macrochir* | 989974 | 204336 | 20.6 |
| *Crocodylus johnsoni* | 1107835 | 226023 | 20.4 |
| *Crocodylus acutus* | 2311771 | 468589 | 20.3 |
| *Carcharhinus leucas* | 3194925 | 636424 | 19.9 |
| *Acipenser medirostris* | 184686 | 35708 | 19.3 |
| *Arius gigas* | 927163 | 178599 | 19.3 |
| *Eleutheronema tetradactylum* | 83164 | 15788 | 19.0 |
| *Pristis pristis* | 1193375 | 222445 | 18.6 |
| *Osteolaemus osborni* | 2134344 | 388391 | 18.2 |
| *Crocodylus moreletii* | 516320 | 92166 | 17.9 |
| *Trichechus senegalensis* | 679978 | 121481 | 17.9 |
| *Probarbus labeamajor* | 59447 | 10560 | 17.8 |
| *Caiman yacare* | 2730413 | 483597 | 17.7 |
| *Crocodylus niloticus* | 17808043 | 3075338 | 17.3 |
| *Heterobranchus longifilis* | 3155678 | 546576 | 17.3 |
| *Clarias gariepinus* | 9627325 | 1617590 | 16.8 |
| *Inia araguaiaensis* | 426343 | 71089 | 16.7 |
| *Osteolaemus tetraspis* | 3181842 | 524297 | 16.5 |
| *Huso huso* | 225613 | 36981 | 16.4 |
| *Salmo trutta* | 6311935 | 1006499 | 15.9 |
| *Lates niloticus* | 5253473 | 822092 | 15.6 |
| *Probarbus jullieni* | 200364 | 30461 | 15.2 |
| *Trionyx triunguis* | 5320848 | 792269 | 14.9 |
| *Salmo salar* | 5924169 | 874356 | 14.8 |
| *Mecistops cataphractus* | 3617566 | 532753 | 14.7 |
| *Caiman latirostris* | 4691656 | 668644 | 14.3 |
| *Acipenser sturio* | 23896 | 3326 | 13.9 |
| *Crocodylus siamensis* | 1771248 | 244117 | 13.8 |
| *Wallago micropogon* | 515629 | 71057 | 13.8 |
| *Catlocarpio siamensis* | 534100 | 71680 | 13.4 |
| *Chrysichthys cranchii* | 930505 | 120905 | 13.0 |
| *Wallago leerii* | 528021 | 68870 | 13.0 |
| *Pelochelys bibroni* | 305452 | 39372 | 12.9 |
| *Crocodylus porosus* | 6335641 | 812918 | 12.8 |
| *Crocodylus rhombifer* | 114852 | 14755 | 12.8 |
| *Pangasius sanitwongsei* | 299487 | 38358 | 12.8 |
| *Hemibagrus wyckioides* | 475447 | 60144 | 12.7 |
| *Hypophthalmichthys molitrix* | 5305300 | 656600 | 12.4 |
| *Orcaella brevirostris* | 787343 | 94364 | 12.0 |
| *Acipenser sinensis* | 102118 | 12121 | 11.9 |
| *Amyda cartilaginea* | 2707564 | 323284 | 11.9 |
| *Neophocaena asiaeorientalis* ssp. *asiaeorientalis* | 102118 | 12121 | 11.9 |
| *Tomistoma schlegelii* | 258900 | 29916 | 11.6 |
| *Acipenser mikadoi* | 29665 | 3343 | 11.3 |
| *Silurus glanis* | 7696293 | 859734 | 11.2 |
| *Chitra chitra* | 388360 | 42933 | 11.1 |
| *Acipenser schrenckii* | 206968 | 22246 | 10.7 |
| *Silurus soldatovi* | 2246081 | 238641 | 10.6 |
| *Acipenser baerii* | 1073267 | 112610 | 10.5 |
| *Orlitia borneensis* | 799675 | 82164 | 10.3 |
| *Acipenser transmontanus* | 536007 | 54862 | 10.2 |
| *Pelochelys cantorii* | 1476768 | 150430 | 10.2 |
| *Acipenser fulvescens* | 2057561 | 195430 | 9.5 |
| *Apalone ferox* | 255285 | 24042 | 9.4 |
| *Pseudoplatystoma corruscans* | 3274795 | 307768 | 9.4 |
| *Ctenopharyngodon idella* | 284831 | 25933 | 9.1 |
| *Hucho taimen* | 12147557 | 1105351 | 9.1 |
| *Wallago attu* | 5476456 | 499767 | 9.1 |
| *Potamotrygon brachyura* | 1409231 | 125147 | 8.9 |
| *Huso dauricus* | 158408 | 13926 | 8.8 |
| *Maccullochella peelii* | 226452 | 19930 | 8.8 |
| *Alligator sinensis* | 113716 | 9858 | 8.7 |
| *Acipenser gueldenstaedtii* | 945921 | 79998 | 8.5 |
| *Acipenser stellatus* | 1001700 | 79226 | 7.9 |
| *Pangasianodon gigas* | 83694 | 6370 | 7.6 |
| *Crocodylus palustris* | 3964340 | 295871 | 7.5 |
| *Labeo rohita* | 4118178 | 303473 | 7.4 |
| *Hypselobarbus mussullah* | 179276 | 12719 | 7.1 |
| *Argyrosomus hololepidotus* | 73870 | 5068 | 6.9 |
| *Nilssonia leithii* | 500974 | 34249 | 6.8 |
| *Lates angustifrons* | 32707 | 2186 | 6.7 |
| *Acipenser oxyrinchus* | 805433 | 48045 | 6.0 |
| *Morone saxatilis* | 708013 | 41601 | 5.9 |
| *Himantura polylepis* | 373212 | 20768 | 5.6 |
| *Acipenser persicus* | 633075 | 34542 | 5.5 |
| *Nilssonia nigricans* | 93833 | 4941 | 5.3 |
| *Chitra indica* | 2596136 | 132731 | 5.1 |
| *Platanista gangetica* ssp. *gangetica* | 368677 | 18714 | 5.1 |
| *Alligator mississippiensis* | 1431075 | 68979 | 4.8 |
| *Platanista gangetica* ssp. *minor* | 68142 | 3173 | 4.7 |
| *Rafetus swinhoei* | 104326 | 4742 | 4.5 |
| *Atractosteus spatula* | 382507 | 16950 | 4.4 |
| *Gavialis gangeticus* | 695665 | 29926 | 4.3 |
| *Acipenser nudiventris* | 521938 | 22120 | 4.2 |
| *Chitra vandijki* | 226869 | 9161 | 4.0 |
| *Hemibagrus maydelli* | 218848 | 8186 | 3.7 |
| *Ictalurus furcatus* | 1147486 | 41791 | 3.6 |
| *Macrochelys temminckii* | 1036973 | 36433 | 3.5 |
| *Scomberomorus sinensis* | 48431 | 1623 | 3.4 |
| *Pylodictis olivaris* | 3056769 | 98801 | 3.2 |
| *Polyodon spathula* | 1016185 | 31721 | 3.1 |
| *Pusa hispida* ssp. *ladogensis* | 17988 | 514 | 2.9 |
| *Scaphirhynchus albus* | 287261 | 8189 | 2.9 |
| *Pusa caspica* | 374357 | 9660 | 2.6 |
| *Luciobarbus esocinus* | 542507 | 7999 | 1.5 |

Table S3.

Number and percentage of species affected within each IUCN Red List threat category (level 1).

| **Threat Category (Level 1)** | **SpRich** | **pcnt** | **FSpRich** | **Fishes%** | **RSpRich** | **Reptiles%** | **MSpRich** | **Mammals%** |
| --- | --- | --- | --- | --- | --- | --- | --- | --- |
| Fishing & harvesting aquatic resources | 66 | 82.5 | 42 | 52.5 | 13 | 16.2 | 11 | 13.8 |
| Dams & water management/use | 52 | 65.0 | 36 | 45.0 | 5 | 6.2 | 11 | 13.8 |
| Agricultural & forestry effluents | 35 | 43.8 | 23 | 28.7 | 3 | 3.8 | 9 | 11.2 |
| Industrial & military effluents | 34 | 42.5 | 22 | 27.5 | 2 | 2.5 | 10 | 12.5 |
| Domestic & urban waste water | 25 | 31.2 | 18 | 22.5 | 2 | 2.5 | 5 | 6.2 |
| Commercial & industrial areas | 19 | 23.8 | 12 | 15.0 | 2 | 2.5 | 5 | 6.2 |
| Shipping lanes | 19 | 23.8 | 12 | 15.0 | NA | NA | 7 | 8.8 |
| Housing & urban areas | 18 | 22.5 | 10 | 12.5 | 3 | 3.8 | 5 | 6.2 |
| Invasive non-native/alien species/diseases | 16 | 20.0 | 14 | 17.5 | 1 | 1.2 | 1 | 1.2 |
| Problematic native species/diseases | 16 | 20.0 | 9 | 11.2 | 2 | 2.5 | 5 | 6.2 |
| Hunting & trapping terrestrial animals | 15 | 18.8 | 1 | 1.2 | 7 | 8.8 | 7 | 8.8 |
| Droughts | 12 | 15.0 | 5 | 6.2 | 1 | 1.2 | 6 | 7.5 |
| Mining & quarrying | 10 | 12.5 | 6 | 7.5 | 2 | 2.5 | 2 | 2.5 |
| Annual & perennial non-timber crops | 10 | 12.5 | 2 | 2.5 | 4 | 5.0 | 4 | 5.0 |
| Marine & freshwater aquaculture | 10 | 12.5 | 6 | 7.5 | 2 | 2.5 | 2 | 2.5 |
| Excess energy | 9 | 11.2 | 5 | 6.2 | NA | NA | 4 | 5.0 |
| Temperature extremes | 8 | 10.0 | 5 | 6.2 | 1 | 1.2 | 2 | 2.5 |
| Logging & wood harvesting | 8 | 10.0 | 5 | 6.2 | 1 | 1.2 | 2 | 2.5 |
| Habitat shifting & alteration | 6 | 7.5 | 2 | 2.5 | 2 | 2.5 | 2 | 2.5 |
| Garbage & solid waste | 6 | 7.5 | 6 | 7.5 | NA | NA | NA | NA |
| Recreational activities | 6 | 7.5 | 3 | 3.8 | NA | NA | 3 | 3.8 |
| Livestock farming & ranching | 5 | 6.2 | 1 | 1.2 | 1 | 1.2 | 3 | 3.8 |
| Tourism & recreation areas | 5 | 6.2 | 2 | 2.5 | 1 | 1.2 | 2 | 2.5 |
| War, civil unrest & military exercises | 5 | 6.2 | NA | NA | NA | NA | 5 | 6.2 |
| Other ecosystem modifications | 4 | 5.0 | NA | NA | 2 | 2.5 | 2 | 2.5 |
| Other threat | 4 | 5.0 | 4 | 5.0 | NA | NA | NA | NA |
| Storms & flooding | 3 | 3.8 | 1 | 1.2 | NA | NA | 2 | 2.5 |
| Oil & gas drilling | 2 | 2.5 | NA | NA | 1 | 1.2 | 1 | 1.2 |
| Roads & railroads | 2 | 2.5 | 1 | 1.2 | NA | NA | 1 | 1.2 |
| Wood & pulp plantations | 2 | 2.5 | NA | NA | 1 | 1.2 | 1 | 1.2 |
| Work & other activities | 2 | 2.5 | NA | NA | 1 | 1.2 | 1 | 1.2 |
| Air-borne pollutants | 1 | 1.2 | 1 | 1.2 | NA | NA | NA | NA |
| Fire & fire suppression | 1 | 1.2 | NA | NA | NA | NA | 1 | 1.2 |
| Introduced genetic material | 1 | 1.2 | NA | NA | 1 | 1.2 | NA | NA |

*Abbreviations:* SpRich, Species richness; FSpRich, Fish species richness; RSpRich, Reptile species richness, MSpRich, Mammal species richness; pcnt, percent; NA, not applicable.

Table S4.

Number and percentage of species affected within each IUCN Red List threat category (level 2).

| **Threat Category (Level 2)** | **SpRich** | **pcnt** | **FSpRich** | **Fishes%** | **RSpRich** | **Reptiles%** | **MSpRich** | **Mammals%** |
| --- | --- | --- | --- | --- | --- | --- | --- | --- |
| Intentional use: (subsistence/small scale) [harvest] | 46 | 57.5 | 34 | 42.5 | 7 | 8.8 | 5 | 6.2 |
| Dams (size unknown) | 44 | 55.0 | 33 | 41.2 | 2 | 2.5 | 9 | 11.2 |
| Type Unknown/ Unrecorded | 33 | 41.2 | 22 | 27.5 | 3 | 3.8 | 8 | 10.0 |
| Unintentional effects: (subsistence/small scale) [harvest] | 31 | 38.8 | 23 | 28.7 | 5 | 6.2 | 3 | 3.8 |
| Intentional use: (large scale) [harvest] | 28 | 35.0 | 23 | 28.7 | 2 | 2.5 | 3 | 3.8 |
| Unintentional effects: (large scale) [harvest] | 28 | 35.0 | 18 | 22.5 | 1 | 1.2 | 9 | 11.2 |
| Soil erosion, sedimentation | 21 | 26.2 | 16 | 20.0 | 2 | 2.5 | 3 | 3.8 |
| Intentional use (species is the target) | 13 | 16.2 | NA | NA | 7 | 8.8 | 6 | 7.5 |
| Unspecified species | 13 | 16.2 | 12 | 15.0 | NA | NA | 1 | 1.2 |
| Sewage | 12 | 15.0 | 9 | 11.2 | NA | NA | 3 | 3.8 |
| Oil spills | 11 | 13.8 | 8 | 10.0 | NA | NA | 3 | 3.8 |
| Motivation Unknown/ Unrecorded | 10 | 12.5 | 6 | 7.5 | NA | NA | 4 | 5.0 |
| Scale Unknown/ Unrecorded | 10 | 12.5 | 6 | 7.5 | 2 | 2.5 | 2 | 2.5 |
| Abstraction of ground water (unknown use) | 8 | 10.0 | 6 | 7.5 | NA | NA | 2 | 2.5 |
| Persecution/ control | 7 | 8.8 | 1 | 1.2 | 3 | 3.8 | 3 | 3.8 |
| Large dams | 6 | 7.5 | 5 | 6.2 | 1 | 1.2 | NA | NA |
| Agro-industry farming | 6 | 7.5 | 1 | 1.2 | 2 | 2.5 | 3 | 3.8 |
| Herbicides and pesticides | 6 | 7.5 | 5 | 6.2 | NA | NA | 1 | 1.2 |
| Thermal pollution | 6 | 7.5 | 5 | 6.2 | NA | NA | 1 | 1.2 |
| Abstraction of surface water (agricultural use) | 4 | 5.0 | 2 | 2.5 | 1 | 1.2 | 1 | 1.2 |
| Nutrient loads | 4 | 5.0 | 4 | 5.0 | NA | NA | NA | NA |
| Named species | 4 | 5.0 | 2 | 2.5 | 2 | 2.5 | NA | NA |
| Noise pollution | 4 | 5.0 | 1 | 1.2 | NA | NA | 3 | 3.8 |
| Run-off | 4 | 5.0 | 3 | 3.8 | NA | NA | 1 | 1.2 |
| Small-holder farming | 4 | 5.0 | 1 | 1.2 | 1 | 1.2 | 2 | 2.5 |
| Nomadic grazing | 3 | 3.8 | NA | NA | NA | NA | 3 | 3.8 |
| Abstraction of surface water (commercial use) | 2 | 2.5 | 1 | 1.2 | NA | NA | 1 | 1.2 |
| Abstraction of surface water (domestic use) | 2 | 2.5 | 1 | 1.2 | NA | NA | 1 | 1.2 |
| Abstraction of surface water (unknown use) | 2 | 2.5 | NA | NA | 2 | 2.5 | NA | NA |
| Agro-industry plantations | 2 | 2.5 | NA | NA | 1 | 1.2 | 1 | 1.2 |
| Unintentional effects (species is not the target) | 2 | 2.5 | NA | NA | 1 | 1.2 | 1 | 1.2 |
| Abstraction of ground water (agricultural use) | 1 | 1.2 | NA | NA | 1 | 1.2 | NA | NA |
| Industrial aquaculture | 1 | 1.2 | NA | NA | 1 | 1.2 | NA | NA |
| Seepage from mining | 1 | 1.2 | 1 | 1.2 | NA | NA | NA | NA |
| Small-holder grazing, ranching or farming | 1 | 1.2 | NA | NA | NA | NA | 1 | 1.2 |
| Trend Unknown/ Unrecorded | 1 | 1.2 | NA | NA | NA | NA | 1 | 1.2 |

*Abbreviations:* SpRich, Species richness; FSpRich, Fish species richness; RSpRich, Reptile species richness, MSpRich, Mammal species richness; pcnt, percent; NA, not applicable.

Table S5.1

Number of all assessed freshwater species with ‘Extant’ and ‘Probably Extant’ records (PRESENCE = 1 and 2, P1&P2) and percentage spatial overlap with the megafauna species ranges.

|  | **Amphibians** | **Birds** | **Decapods** | **Fishes** | **Mammals** | **Molluscs** | **Odonata** | **Plants** | **Turtles** | **All Taxa** |
| --- | --- | --- | --- | --- | --- | --- | --- | --- | --- | --- |
| A: Number of assessed species | 4375 | 2283 | 2630 | 7620 | 150 | 3495 | 2784 | 1880 | 183 | 25400 |
| B: Number of assessed species with P1&2 records | 3972 | 2016 | 2441 | 5956 | 122 | 1836 | 1313 | 1201 | 167 | 19024 |
| C: Number of assessed species with P1&2 records in the megafauna range | 3739 | 1970 | 2266 | 5648 | 119 | 1398 | 1285 | 1094 | 160 | 17679 |
| C as % of A | 85 | 86 | 86 | 74 | 79 | 40 | 46 | 58 | 87 | 70 |
| C as % of B | 94 | 98 | 93 | 95 | 98 | 76 | 98 | 91 | 96 | 93 |

*Note:* Decapods comprise crabs, crayfish and shrimps.

Table S5.2.

Number of all assessed threatened (i.e. CR, EN, VU) freshwater species with ‘Extant’ and ‘Probably Extant’ records (PRESENCE = 1 and 2, P1&P2) and percentage spatial overlap with the megafauna species ranges.

|  | **Amphibians** | **Birds** | **Decapods** | **Fishes** | **Mammals** | **Molluscs** | **Odonata** | **Plants** | **Turtles** | **All Taxa** |
| --- | --- | --- | --- | --- | --- | --- | --- | --- | --- | --- |
| A: Number of threatened species | 1171 | 226 | 483 | 1824 | 54 | 1021 | 265 | 319 | 102 | 5465 |
| B: Number of threatened species with P1&2 records | 1071 | 204 | 421 | 1237 | 43 | 623 | 72 | 203 | 92 | 3966 |
| C: Number of threatened species with P1&2 records in megafauna range | 960 | 182 | 387 | 1058 | 41 | 364 | 64 | 148 | 89 | 3293 |
| C as % of A | 82 | 81 | 80 | 58 | 76 | 36 | 24 | 46 | 87 | 60 |
| C as % of B | 90 | 89 | 92 | 86 | 95 | 58 | 89 | 73 | 97 | 83 |

*Note:* Decapods comprise crabs, crayfish and shrimps.

Table S6.

Megafauna species representation with international conventions; Convention on Migratory Species (CMS) and Convention on International Trade in Endangered Species of Wild Fauna and Flora (CITES).

| **Binomial** | **CMS Appendices** | | **CITES Appendices** | | |
| --- | --- | --- | --- | --- | --- |
|  | I | II | I | II | III |
| *Acipenser gueldenstaedtii* |  | 1999 |  | 1998 |  |
| *Acipenser mikadoi* |  | 1999 |  | 1998 |  |
| *Acipenser nudiventris* |  | 1999 |  | 1998 |  |
| *Acipenser persicus* |  | 1999 |  | 1998 |  |
| *Acipenser schrenckii* |  | 1999 |  | 1998 |  |
| *Acipenser sinensis* |  | 1999 |  | 1998 |  |
| *Acipenser stellatus* | 1985 | 1999 |  | 1998 |  |
| *Acipenser sturio* | 2005 | 1999 | 1983 |  |  |
| *Catlocarpio siamensis* |  |  |  |  |  |
| *Huso dauricus* |  | 1999 |  | 1998 |  |
| *Huso huso* |  | 1979 |  | 1998 |  |
| *Maccullochella peelii* |  |  |  |  |  |
| *Pangasianodon gigas* | 1979 |  | 1975 |  |  |
| *Pangasius sanitwongsei* |  |  |  |  |  |
| *Pristis pectinata* | 2014 | 2014 | 2007 |  |  |
| *Pristis pristis* | 2014 | 2014 | 2007 |  |  |
| *Psephurus gladius* |  | 1999 |  | 1998 |  |
| *Lipotes vexillifer* |  |  | 1979 |  |  |
| *Alligator sinensis* |  |  | 1975 |  |  |
| *Chitra chitra* |  |  | 2013 |  |  |
| *Chitra vandijki* |  |  | 2013 |  |  |
| *Crocodylus intermedius* |  |  | 1975 |  |  |
| *Crocodylus rhombifer* |  |  | 1975 |  |  |
| *Crocodylus siamensis* |  |  | 1975 |  |  |
| *Gavialis gangeticus* | 1979 |  | 1975 |  |  |
| *Mecistops cataphractus* |  |  | 1992 |  |  |
| *Nilssonia leithii* |  |  |  | 2013 |  |
| *Nilssonia nigricans* |  |  | 1975 |  |  |
| *Orlitia borneensis* |  |  |  | 2013 |  |
| *Podocnemis expansa* | 1979 | 1979 |  | 1975 |  |
| *Rafetus swinhoei* |  |  |  | 2013 |  |
|  |  |  |  |  |  |
| *Acipenser baerii* |  | 1999 |  | 1998 |  |
| *Argyrosomus hololepidotus* |  |  |  |  |  |
| *Himantura polylepis* |  |  |  |  |  |
| *Hucho hucho* |  |  |  |  |  |
| *Hypselobarbus mussullah* |  |  |  |  |  |
| *Lates angustifrons* |  |  |  |  |  |
| *Probarbus jullieni* |  |  | 1975 |  |  |
| *Probarbus labeamajor* |  |  |  |  |  |
| *Scaphirhynchus albus* |  |  |  | 1998 |  |
| *Tor putitora* |  |  |  |  |  |
| *Choeropsis liberiensis* |  |  |  | 1975 |  |
| *Kobus megaceros* |  |  |  |  |  |
| *Platanista gangetica* ssp. *gangetica* | 2002 | 1991 | 1981 |  |  |
| *Platanista gangetica* ssp. *minor* | 2002 | 1999 | 1981 |  |  |
| *Pusa caspica* |  |  |  |  |  |
| *Chitra indica* |  |  |  | 2013 |  |
| *Pelochelys cantorii* |  |  |  | 2003 |  |
|  |  |  |  |  |  |
| *Hucho taimen* |  |  |  |  |  |
| *Luciobarbus esocinus* |  |  |  |  |  |
| *Megalops atlanticus* |  |  |  |  |  |
| *Polyodon spathula* |  |  |  | 1998 |  |
| *Hippopotamus amphibius* |  |  |  | 1995 |  |
| *Neophocaena asiaeorientalis* ssp. *asiaeorientalis* |  | 1979*^a^ |  |  |  |
| *Orcaella brevirostris* | 2009 | 1991^b^ | 2005 |  |  |
| *Trichechus inunguis* |  | 2002 | 1975 |  |  |
| *Trichechus manatus* | 1999 | 1999 | 1975 |  |  |
| *Trichechus senegalensis* | 2009 | 2002^c^ | 2013 |  |  |
| *Amyda cartilaginea* |  |  |  | 2005 |  |
| *Crocodylus acutus* |  |  | 2005 | 2005 |  |
| *Crocodylus palustris* |  |  | 1975 |  |  |
| *Macrochelys temminckii* |  |  |  |  | 2006 |
| *Osteolaemus tetraspis* |  |  | 1992 |  |  |
| *Pelochelys bibroni* |  |  |  | 2003 |  |
| *Tomistoma schlegelii* |  |  | 1975 |  |  |
| *Trionyx triunguis* |  |  |  | 2016 |  |
|  |  |  |  |  |  |
| *Acipenser medirostris* |  | 1999 |  | 1998 |  |
| *Acipenser oxyrinchus* |  |  |  | 1998 |  |
| *Arius gigas* |  |  |  |  |  |
| *Carcharhinus leucas* |  |  |  |  |  |
| *Hypophthalmichthys molitrix* |  |  |  |  |  |
| *Wallago attu* |  |  |  |  |  |
|  |  |  |  |  |  |
| *Acipenser fulvescens* |  | 1979 |  | 1998 |  |
| *Acipenser transmontanus* |  |  |  | 1998 |  |
| *Chrysichthys cranchii* |  |  |  |  |  |
| *Clarias gariepinus* |  |  |  |  |  |
| *Hemibagrus maydelli* |  |  |  |  |  |
| *Hemibagrus wyckioides* |  |  |  |  |  |
| *Heterobranchus longifilis* |  |  |  |  |  |
| *Hydrocynus goliath* |  |  |  |  |  |
| *Ictalurus furcatus* |  |  |  |  |  |
| *Labeo rohita* |  |  |  |  |  |
| *Lates niloticus* |  |  |  |  |  |
| *Morone saxatilis* |  |  |  |  |  |
| *Salmo salar* |  |  |  |  |  |
| *Salmo trutta* |  |  |  |  |  |
| *Silurus glanis* |  |  |  |  |  |
| *Hydrochoerus hydrochaeris* |  |  |  |  |  |
| *Kobus leche* |  |  |  | 1979 |  |
| *Phoca vitulina mellonae* |  |  |  |  |  |
| *Pusa sibirica* |  |  |  |  |  |
| *Tragelaphus spekii* |  |  |  |  |  |
| *Alligator mississippiensis* |  |  |  | 1979 |  |
| *Apalone ferox* |  |  |  |  | 2016 |
| *Caiman crocodilus* |  |  |  | 1977 |  |
| *Caiman latirostris* |  |  | 1997 | 1997 |  |
| *Caiman yacare* |  |  |  | 1977 |  |
| *Crocodylus johnsoni* |  |  |  | 1977 |  |
| *Crocodylus moreletii* |  |  | 2010 | 2010 |  |
| *Crocodylus niloticus* |  |  | 2010 | 2010 |  |
| *Crocodylus porosus* |  | 1979 | 1995 | 1995 |  |
| *Melanosuchus niger* |  |  | 2007 | 2007 |  |
| *Paleosuchus palpebrosus* |  |  |  | 1977 |  |
|  |  |  |  |  |  |
| *Arapaima gigas* |  |  |  | 1975 |  |
| *Potamotrygon brachyura* |  |  |  |  | 2017 |
| *Scomberomorus sinensis* |  |  |  |  |  |
| *Wallago micropogon* |  |  |  |  |  |
| *Inia geoffrensis* |  | 1991 |  | 2003 |  |
| *Sotalia fluviatilis* |  | 1979 | 1979 |  |  |
|  |  |  |  |  |  |
| *Arapaima agassizii* |  |  |  |  |  |
| *Arapaima leptosoma* |  |  |  |  |  |
| *Arapaima mapae* |  |  |  |  |  |
| *Atractosteus spatula* |  |  |  |  |  |
| *Brachyplatystoma filamentosum* |  |  |  |  |  |
| *Colossoma macropomum* |  |  |  |  |  |
| *Ctenopharyngodon idella* |  |  |  |  |  |
| *Eleutheronema tetradactylum* |  |  |  |  |  |
| *Lates calcarifer* |  |  |  |  |  |
| *Oncorhynchus tshawytscha* |  |  |  |  |  |
| *Polydactylus macrochir* |  |  |  |  |  |
| *Pseudoplatystoma corruscans* |  |  |  |  |  |
| *Pseudoplatystoma fasciatum* |  |  |  |  |  |
| *Pylodictis olivaris* |  |  |  |  |  |
| *Silurus soldatovi* |  |  |  |  |  |
| *Wallago leerii* |  |  |  |  |  |
| *Zungaro zungaro* |  |  |  |  |  |
| *Inia araguaiaensis* |  |  |  | 2014 |  |
| *Inia boliviensis* |  |  |  | 2003 |  |
| *Pusa hispida* ssp. *ladogensis* |  |  |  |  |  |
| *Pusa hispida* ssp. *saimensis* |  |  |  |  |  |
| *Eunectes murinus* |  |  |  | 1977 |  |
| *Osteolaemus osborni* |  |  | 1992 |  |  |

*Note:* *Parent species; ^a^ASCOBANS; ^b^CMS, Pacific Islands Cetaceans; ^c^CMS, Western African Aquatic Mammals

Table S7.

Weight references for each megafauna species.

| **Binomial** | **Weight (kg)** | **Reference** |
| --- | --- | --- |
| *Acipenser baerii* | 210 | Kottelat and Freyhof (2007) |
| *Acipenser fulvescens* | 125 | Carlander (1969) |
| *Acipenser gueldenstaedtii* | 115 | Birstein (1993) |
| *Acipenser medirostris* | 159 | Peterson et al. (1999) |
| *Acipenser mikadoi* | 80 | Shilin (1995), Shmigirilov et al. (2007) |
| *Acipenser nudiventris* | 80 | Rochard et al. (1991) |
| *Acipenser oxyrinchus* | 368 | Mangin (1964) |
| *Acipenser persicus* | 70 | Vecsei and Artyukhin (2001) |
| *Acipenser schrenckii* | 190 | Krykhtin and Svirskii (1997) |
| *Acipenser sinensis* | 600 | Zhang (2001) |
| *Acipenser stellatus* | 80 | Frimodt (1995) |
| *Acipenser sturio* | 400 | Muus (1968) |
| *Acipenser transmontanus* | 816 | Lamb (1986) |
| *Alligator mississippiensis* | 473 | Lobaina (2014) |
| *Alligator sinensis* | 38 | Thorbjarnarson J. et al. (2001) |
| *Andrias davidianus* | 50 | Wang et al. (2004) |
| *Arapaima* spp. | 200 | Castello and Stewart (2010) |
| *Argyrosomus hololepidotus* | 71 | Trewavas (1977) |
| *Arius gigas* | 50 | Ita (1984) |
| *Atractosteus spatula* | 137 | Stone (2007) |
| *Brachyplatystoma filamentosum* | 200 | Boujard (1997) |
| *Caiman crocodilus* | 58 | Ojasti (1996) |
| *Caiman latirostris* | 62 | Ferraz et al. (2005) |
| *Caiman yacare* | 58 | Ojasti (1996) |
| *Carcharhinus leucas* | 238 | Wintner et al. (2002) |
| *Catlocarpio siamensis* | 300 | Roberts and Warren (1994) |
| *Chitra chitra* | 202 | Kitimasak et al. (2005) |
| *Chitra indica* | 57 | Das I. and Singh (2014) |
| *Chitra vandijki* | 100 | Platt et al. (2009) |
| *Choeropsis liberiensis* | 275 | Boisserie (2007) |
| *Chrysichthys cranchii* | 135 | Risch and Bagridae (1986) |
| *Clarias gariepinus* | 60 | Robins (1991) |
| *Crocodylus acutus* | 173 | Lobaina (2014) |
| *Crocodylus intermedius* | 380 | Lobaina (2014) |
| *Crocodylus johnsoni* | 31 | Walsh (1989) |
| *Crocodylus moreletii* | 58 | Lobaina (2014) |
| *Crocodylus niloticus* | 200 | Hutton (1987) |
| *Crocodylus palustris* | 200 | Lobaina (2014) |
| *Crocodylus porosus* | 2000 | Ogamba and Abowei (2012) |
| *Crocodylus rhombifer* | 215 | Lobaina (2014) |
| *Crocodylus siamensis* | 50 | Daltry et al. (2003) |
| *Ctenopharyngodon idella* | 50 | Cudmore and Mandrak (2004) |
| *Eleutheronema tetradactylum* | 145 | Grant (1978) |
| *Eunectes murinus* | 200 | Miller et al. (2004) |
| *Gavialis gangeticus* | 160 | Stevenson and Whitaker (2010) |
| *Hemibagrus maydelli* | 58 | Jayaram (1995) |
| *Hemibagrus wyckioides* | 80 | Roberts (1993), Hee and Rainboth (1999) |
| *Heterobranchus longifilis* | 55 | Skelton (2001) |
| *Himantura chaophraya* | 600 | Last and Stevens (2009) |
| *Hippopotamus amphibius* | 4500 | Coughlin and Fish (2009) |
| *Hucho hucho* | 52 | Nikolskii (1957) |
| *Hucho taimen* | 105 | Kottelat and Freyhof (2007)F |
| *Huso dauricus* | 1000 | Krykhtin and Svirskii (1997) |
| *Huso huso* | 3200 | Kottelat and Freyhof (2007) |
| *Hydrochoerus hydrochaeris* | 81 | Ferraz et al. (2005) |
| *Hydrocynus goliath* | 50 | Robins (1991) |
| *Hypophthalmichthys molitrix* | 50 | Krykhtin and Svirskii (1997) |
| *Hypselobarbus mussullah* | 90 | Talwar and Jhingran (1991) |
| *Ictalurus furcatus* | 68 | Frimodt (1995) |
| *Inia araguaiaensis* | 207 | Da Silva (2009) |
| *Inia boliviensis* | 207 | Da Silva (2009) |
| *Inia geoffrensis* | 207 | Da Silva (2009) |
| *Kobus leche* | 128 | Estes (1991) |
| *Kobus megaceros* | 113 | Bercovitch et al. (2009) |
| *Labeo rohita* | 45 | Frimodt (1995) |
| *Lates angustifrons* | 100 | Stone (2007) |
| *Lates calcarifer* | 60 | Larson (2001) |
| *Lates niloticus* | 200 | Ribbink (1987) |
| *Lipotes vexillifer* | 237 | Zhou K et al. (1977), Zhou Kaiya (1986) |
| *Luciobarbus esocinus* | 140 | Robins (1991) |
| *Maccullochella peelii* | 113 | Rowland (1989) |
| *Macrochelys temminckii* | 90 | Jensen and Birkhead (2003) |
| *Mecistops cataphractus* | 230 | Lobaina (2014) |
| *Megalops atlanticus* | 161 | Claro (1994) |
| *Melanosuchus niger* | 400 | Da Silveira et al. (2010), Thorbjarnarson John B (2010), Cardoso et al. (2012) |
| *Morone saxatilis* | 57 | Peterson et al. (1999) |
| *Neophocaena asiaeorientalis asiaeorientalis* | 61 | Yang et al. (2008) |
| *Oncorhynchus tshawytscha* | 61 | Morrow (1980) |
| *Orcaella brevirostris* | 133 | Arnold and Heinsohn (1996) |
| *Orlitia borneensis* | 50 | Halliday and Adler (2002) |
| *Osteolaemus osborni* | 80 | Lobaina (2014) |
| *Osteolaemus tetraspis* | 80 | Lobaina (2014) |
| *Paleosuchus palpebrosus* | 37 | Campos et al. (2010) |
| *Pangasianodon gigas* | 350 | Kottelat (2001) |
| *Pangasius sanitwongsei* | 300 | Roberts and Vidthayanon (1991) |
| *Pelochelys bibroni* | 120 | Bonin et al. (2006) |
| *Pelochelys cantorii* | 43 | Das I (2008) |
| *Phoca vitulina* spp. *mellonae* | 70 | Smith (2000) |
| *Platanista gangetica* | 108 | Jefferson TA et al. (1994) |
| *Platanista gangetica* ssp. *minor* | 110 | Waqas et al. (2012) |
| *Podocnemis expansa* | 90 | Clauson et al. (1989) |
| *Polydactylus macrochir* | 30 | Motomura et al. (2000) |
| *Polyodon spathula* | 90 | McClane (1978) |
| *Potamotrygon brachyura* | 120 | Franco et al. (2011) |
| *Pristis pristis* | 600 | Stehman (1981) |
| *Pristis pectinata* | 350 | Stehman (1981) |
| *Probarbus jullieni* | 70 | Roberts and Baird (1995) |
| *Probarbus labeamajor* | 70 | Roberts and Warren (1994) |
| *Psephurus gladius* | 300 | Mims and Georgi (1993) |
| *Pseudoplatystoma corruscans* | 100 | Tavares (1997) |
| *Pseudoplatystoma fasciatum* | 70 | Le Bail et al. (2000) |
| *Pusa caspica* | 66 | Ikemoto et al. (2004) |
| *Pusa hispida* ssp. *ladogensis* | 70 | Popov (1979) |
| *Pusa hispida* ssp. *saimensis* | 100 | Sipilä and Hyvärinen (1998) |
| *Pusa sibirica* | 90 | Popov (1982) |
| *Pylodictis olivaris* | 50 | Brown et al. (2005) |
| *Rafetus swinhoei* | 115 | Jian et al. (2013) |
| *Salmo salar* | 46 | Dymond (1963) |
| *Salmo trutta* | 50 | Muus and Dahlstrom (1981) |
| *Scaphirhynchus albus* | 130 | Rochard et al. (1991) |
| *Scomberomorus sinensis* | 131 | Collette et al. (2011) |
| *Silurus glanis* | 306 | Frimodt (1995) |
| *Silurus soldatovi* | 40 | Berg (1962) |
| *Sotalia fluviatilis* | 40 | Jefferson Thomas A (1993) |
| *Tomistoma schlegelii* | 210 | Lobaina (2014) |
| *Tor putitora* | 54 | Rahman (1989) |
| *Tragelaphus spekii* | 100 | Estes (1991) |
| *Trichechus inunguis* | 480 | Amaral et al. (2010) |
| *Trichechus manatus* | 1500 | Spellman (2014) |
| *Trichechus senegalensis* | 500 | Dodman et al. (2012) |
| *Trionyx triunguis* | 45 | Rozner and Shaines (2010) |
| *Wallago attu* | 45 | Achakzai et al. (2013) |
| *Wallago leerii* | 86 | Roberts (1989) |
| *Wallago micropogon* | 96 | Roberts (1993) |
| *Zungaro zungaro* | 50 | Le Bail et al. (2000) |

References to Table S7

Achakzai W, Baloch W, Saddozai S, Memon N. 2013. Length‐weight relationships (LWRs) of Wallago attu (Bloch and Schneider) from Manchar Lake Jamshoro, Sindh, Pakistan. Journal of Applied Ichthyology 29:1172-1172.

Amaral RS, da Silva VM, Rosas FC. 2010. Body weight/length relationship and mass estimation using morphometric measurements in Amazonian manatees Trichechus inunguis (Mammalia: Sirenia). Marine Biodiversity Records 3:e105.

Arnold PW, Heinsohn GE. 1996. Phylogenetic status of the Irrawaddy dolphin Orcaella brevirostris (Owen in Gray): a cladistic analysis. Memoirs-Queensland Museum 39:141-204.

Bercovitch FB, Loomis CP, Rieches RG. 2009. Age-specific changes in reproductive effort and terminal investment in female Nile lechwe. Journal of Mammalogy 90:40-46.

Berg LS. 1962. Freshwater fishes of the USSR and adjacent countries:(Ryby presnykh vod SSSR i sopredelʹnykh stran). Israel Program for Scientific Translations.

Birstein VJ. 1993. Sturgeons and Paddlefishes - Threatened Fishes in Need of Conservation. Conservation Biology 7:773-787.

Boisserie JR. 2007. Family Hippopotamidae. Pages 106-119 in Prothero DR, Foss SE, eds. The Evolution of Artiodactyls. Johns Hopkins University Press.

Bonin F, Devaux B, Dupré A. 2006. Turtles of the World. Johns Hopkins University Press.

Boujard T, Pascal M, Meunier FJ, Le Bail PY 1997. Poissons de Guyane: Guide écologique de l'Approuague et de la réserve des Nouragues. Institut National de la Recherche Agronomique.

Brown JJ, Perillo J, Kwak TJ, Horwitz RJ. 2005. Implications of Pylodictis olivaris (flathead catfish) introduction into the Delaware and Susquehanna drainages. Northeastern Naturalist 12:473-484.

Campos Z, Sanaiotti T, Magnusson WE. 2010. Maximum size of dwarf caiman, Paleosuchus palpebrosus (Cuvier, 1807), in the Amazon and habitats surrounding the Pantanal, Brazil. Amphibia-Reptilia 31:439-442.

Cardoso A, de Souza A, Menezes R, Pereira W, Tortelly R. 2012. Gastric Lesions in Free-Ranging Black Caimans (Melanosuchus niger) Associated With Brevimulticaecum Species. Veterinary Pathology 50:582-584.

Carlander KD. 1969. Handbook of freshwater fishery biology. Iowa State University Press.

Castello L, Stewart DJ. 2010. Assessing CITES non‐detriment findings procedures for Arapaima in Brazil. Journal of Applied Ichthyology 26:49-56.

Claro A. 1994. Características generales de la ictiofauna. Pages 55-70 in Claro A, ed. Ecología de los peces marinos de Cuba, Instituto de Oceanología Academia de Ciencias de Cuba and Centro de Investigaciones de Quintana Roo.

Clauson B, Timm R, Albuja Viteri LH. 1989. Siona Hunting Techniques for the Larger Aquatic Vertebrates in Amazonian Ecuador. Studies on Neotropical Fauna 24:1-7.

Collette B, Chang SK, Di Natale A, Fox W, Juan Jorda M, Nelson R, Uozumi Y. 2011. Scomberomorus sinensis. The IUCN Red List of Threatened Species 2011. Report no. e.T170346A6760678.

Coughlin BL, Fish FE. 2009. Hippopotamus Underwater Locomotion: Reduced-Gravity Movements for a Massive Mammal. Journal of Mammalogy 90:675-679.

Cudmore B, Mandrak NE. 2004. Biological synopsis of grass carp (Ctenopharyngodon idella). Report no.

Da Silva V. 2009. Amazon river dolphin Inia geoffrensis. Pages 26-28 in Perrin W, Würsig B, Thewissen J, eds. Encyclopedia of marine mammals. Academic Press.

Da Silveira R, Ramalho EE, Thorbjarnarson JB, Magnusson WE. 2010. Depredation by Jaguars on Caimans and Importance of Reptiles in the Diet of Jaguar. Journal of Herpetology 44:418-424.

Daltry J, Chheang D, Em P, Poeung M, Sam H, Sorn P, Tan T, Simpson B. 2003. Status of the Siamese Crocodile in the Central Cardamom Mountains, Southwest Cambodia. Cambodian Crocodile Conservation Programme, Fauna & Flora Internation and Department of Forestry and Wildlife, Phnom Penh.

Das I. 2008. Pelochelys cantorii Gray 1864–Asian giant softshell turtle. Chelonian Research Monographs 5:011.1 - 011.6.

Das I, Singh S. 2014. Chitra indica (Gray 1830)–narrow-headed softshell turtle. Chelonian Research Monographs 5:027.1-027.7.

Dodman T, Dagou Diop M, Beye C. 2012. The West African manatee: A flagship wetland species in decline. Report no. CMS Technical Series No. 26.

Dymond JR. 1963. Family salmonidae.Fishes of the Western North Atlantic. Memoir of the Sears Foundation for Marine Research:457-502.

Estes R. 1991. The behavior guide to African mammals. University of California Press

Ferraz KMPMd, Bonach K, Verdade LM. 2005. Relationship between body mass and body length in capybaras (Hydrochoerus hydrochaeris). Biota Neotropica 5:197-200.

Franco MCO, Canziani GV, Charvet P. 2011. Record of the freshwater stingrays Potamotrygon brachyura and P. motoro (Potamotrygonidae) in the lower Uruguay River, South America. Acta Amazonica 42.

Frimodt C. 1995. Multilingual illustrated guide to the world's commercial coldwater fish. Fishing News Books Ltd.

Grant EM. 1978. Guide to fishes. Department of Harbours and Marine, Brisbane.

Halliday T, Adler K. 2002. The New Encyclopaedia of Reptiles and Amphibians. Oxford University Press.

Hee NH, Rainboth WJ. 1999. The bagrid catfish genus Hemibagrus (Teleostei : Siluriformes) in central Indochina with a new species from the Mekong River. Raffles Bulletin of Zoology 47:555-576.

Hutton JM. 1987. Morphometrics and Field Estimation of the Size of the Nile Crocodile. African Journal of Ecology 25:225-230.

Ikemoto T, Kunito T, Watanabe I, Yasunaga G, Baba N, Miyazaki N, Petrov EA, Tanabe S. 2004. Comparison of trace element accumulation in Baikal seals (Pusa sibirica), Caspian seals (Pusa caspica) and northern fur seals (Callorhinus ursinus). Environmental Pollution 127:83-97.

Ita EO. 1984. Kainji (Nigeria). Pages 43-103. Kapetsky JM, Tomi P. eds. Status of African reservoir fisheries. Food and Agriculture Organisation of United Nations

Jayaram KC. 1995. The Krishna River System: A Bioresources Study. Calcutta, India. Records of Zoological Society of India. Report no. Occasional Paper No. 160.

Jefferson T, Leatherwood S, Webber M. 1994. Marine Mammal Science: FAO Species Identification Guide. United Nations Environment Programme and Food and Agriculture Organisation of United Nations.

Jefferson TA. 1993. FAO species identification guide. Marine mammals of the world. Food and Agriculture Organisation of United Nations.

Jensen JB, Birkhead WS. 2003. Distribution and status of the alligator snapping turtle (Macrochelys temminckii) in Georgia. Southeastern Naturalist 2:25-34.

Wang J, Shi H, Wen Cheng, Han L. 2013. Habitat Selection and Conservation Suggestions for the Yangtze Giant Softshell Turtle (Rafetus swinhoei) in the Upper Red River, China. Chelonian Conservation and Biology 12:177-184.

Kitimasak W, Thirakhupt K, Boonyaratpalin S, Moll DL. 2005. Distribution and population status of the narrow-headed softshell turtle Chitra spp. in Thailand. Natural History 5:31-42.

Kottelat M. 2001. Fishes of Laos. WHT Publications Ltd.

Kottelat M, Freyhof Jr. 2007. Handbook of European freshwater fishes. Publications Kottelat.

Krykhtin ML, Svirskii VG. 1997. Endemic sturgeons of the Amor River: Kaluga, Huso dauricus, and Amur sturgeon, Acipenser schrenckii. Environmental Biology of Fishes 48:231-239.

Lamb A. 1986. Coastal fishes of the Pacific Northwest. Harbour Publishing Company.

Larson H. 2001. Freshwater fishes of the Northern Territory. Northern Territory Government Printing Office.

Last PR, Stevens JD. 2009. Sharks and rays of Australia. CSIRO Publishing.

Le Bail P-Y, Keith P, Planquette P. 2000. Atlas des poissons d'eau douce de Guyane.Tome 2, fasc. 2: Siluriformes. Muséum national d'histoire naturelle.

Lobaina I. 2014. Evolution of maternal investment strategies for the order Crocodylia. Undergraduate's Thesis. University of South Florida St. Petersburg.

Mangin E. 1964. Croissance en Longueur de Trois Esturgeons d'Amerique du Nord: Acipenser oxyrhynchus, Mitchill, Acipenser fulvescens, Rafinesque, et Acipenser brevirostris LeSueur. Verh. Int. Ver. Limnology 15:968-974.

McClane AJ. 1978. McClane's field guide to freshwater fishes of North America. Henry Holt.

Miller DL, Radi ZA, Stiver SL, Thornhill TD. 2004. Cutaneous and pulmonary mycosis in green anacondas (Euncectes murinus). Journal of Zoo and Wildlife Medicine 35:557-561.

Mims S, Georgi T. 1993. The Chinese paddlefish: biology, life history and potential for cultivation. World Aquaculture 24:46-46.

Morrow JE. 1980. The freshwater fishes of Alaska. Alaska Northwest Publishing Company.

Motomura H, Iwatsuki Y, Kimura S, Yoshino T. 2000. Redescription of Polydactylus macrochir (Gunther, 1867), a senior synonym of P. sheridani (Macleay, 1884) (Perciformes : Polynemidae). Ichthyological research 47:327-333.

Muus BJ. 1968. Süßwasserfische. BLV

Muus BJ, Dahlstrom P. 1981. Guide des poissons d'eau douce et pêche. Delachaux & Niestlé.

Nikolskii G. 1957. Spezielle Fischkunde (translated from Russian). VEB Deutscher Verlag der Wissenschaften.

Ogamba E, Abowei J. 2012. Some Aquatic Reptiles in Culture Fisheries Management. International Journal of Fishes and Aquatic Sciences 1:5-15.

Ojasti J. 1996. Wildlife utilization in Latin America: current situation and prospects for sustainable management. Food and Agriculture Organization of the United Nations.

Peterson RT, Eschmeyer WN, Herald ES. 1999. A field guide to Pacific coast fishes: North America. Houghton Mifflin Harcourt.

Platt SG, Platt K, Win KK, Rainwater TR. 2009. Chitra vandijki McCord and Pritchard 2003 – Burmese Narrow-Headed Softshell Turtle. Chelonian Research Monographs 5:074.1-074.7.

Popov L. 1979. Ladoga Seal. Pages 70-71 in Food and Agriculture Organization of the United Nations, Working Party on Marine Mammals, eds. Mammals in the Seas - pinniped species summaries and report on sirenians vol. 2. Food and Agriculture Organisation of United Nations.

Popov L. 1982. Baikal Seal. Pages 378 in Food and Agriculture Organization of the United Nations, Working Party on Marine Mammals, eds. Mammals in the Seas - Small Cetaceans, Seals, Sirenians, and Otters vol. 4. Food and Agriculture Organisation of United Nations.

Rahman A. 1989. Freshwater fishes of Bangladesh. Zoological Society of Bangladesh, Department of Zoology, University of Dhaka.

Ribbink AJ. 1987. African lakes and their fishes: conservation scenarios and suggestions. Environmental Biology of Fishes 19:3-26.

Risch L, Bagridae I. 1986. Check-list of the Freshwater Fishes of Africa. Pages 2-35 in Daget J, Gosse J-P, Thys van den Audenaerde DFE, eds. Check-list of the freshwater fishes of Africa (cloffa), vol. 2. Institut Royal des Sciences Naturelles de Belgique.

Roberts TR. 1989. The freshwater fishes of western Borneo (Kalimantan Barat, Indonesia). California Academy of Science.

Roberts TR. 1993. Artisanal fisheries and fish ecology below the great waterfalls of the Mekong River in southern Laos. Natural History Bulletin of the Siam Society 41:31-62.

Roberts TR, Baird IG. 1995. Traditional fisheries and fish ecology on the Mekong River at Khone Waterfalls in southern Laos. Natural History Bulletin of the Siam Society 43:219-262.

Roberts TR, Vidthayanon C. 1991. Systematic revision of the Asian catfish family Pangasiidae, with biological observations and descriptions of three new species. Proceedings of the Academy of Natural Sciences of Philadelphia:97-143.

Roberts TR, Warren TJ. 1994. Observations on fishes and fisheries in southern Laos and northeastern Cambodia, October 1993-February 1994. Natural History Bulletin of the Siam Society 42:87-115.

Robins CR. 1991. World fishes important to North Americans: exclusive of species from the continental waters of the United States and Canada. American Fisheries Society.

Rochard E, Williot P, Castelnaud G, Lepage M. 1991. Eléments de systématique et de biologie des populations sauvages d’esturgeons. Pages 475-507 in Williot P, ed. Acipenser, Actes du Premier Colloque International sur l’Esturgeon. Cemagref.

Rowland S. 1989. Aspects of the history and fishery of the Murray cod, Maccullochella peeli (Mitchell)(Percichthyidae). Proceedings of the Linnean Society of New South Wales 111:201-213.

Rozner O, Shaines U. 2010. Research report on the Nile Soft shell turtle (Trionyx triunguis) in Nahal Alexander. Department of Biology and Environmental Evolution, Haifa University.

Shilin N. 1995. Programme for conservation of Acipenser medirostris mikadoi in the Russian Far East. Pages 262-267. Proceedings of the international sturgeon symposium. VNIRO Publishing.

Shmigirilov AP, Mednikova AA, Israel JA. 2007. Comparison of biology of the Sakhalin sturgeon, Amur sturgeon, and kaluga from the Amur River, Sea of Okhotsk, and Sea of Japan biogeographic Province. Environmental Biology of Fishes 79:383-395.

Sipilä T, Hyvärinen H. 1998. Status and biology of Saimaa (Phoca hispida saimensis) and Ladoga (Phoca hispida ladogensis) ringed seals. NAMMCO Scientific Publications 1:83-99.

Skelton PH. 2001. A complete guide to the freshwater fishes of southern Africa. Southern Book Publishers.

Smith RJ. 2000. The Lacs Des Loups Marin Harbour Seal, Phoca Vitulina Mellonae Doutt 1942: Ecology of an Isolated Population. University of Guelph.

Spellman AC. 2014. Physiological Constraints on Warm-water Habitat Site Selection and Utilization by the Florida Manatee (Trichechus manatus latirostris) in East Central Florida. University of Central Florida.

Stehman M. 1981. Pristidae in Fischer W, Bianchi G, Scott WB, eds. FAO Species Identification Sheets for Fishery Purposes Eastern Central Atlantic. Rome: Food and Agriculture Organisation of United Nations.

Stevenson C, Whitaker R. 2010. Indian Gharial Gavialis gangeticus. Pages 139-143. in IUCN/SSC Crocodile Specialist Group, ed. Crocodiles: Status Survey and Conservation Action Plan. IUCN.

Stone R. 2007. The last of the leviathans. Science 316:1684-1688.

Talwar PK, Jhingran AG. 1991. Inland fishes of India and adjacent countries. Oxford and IBH Publication Co.

Tavares M. 1997. O surubim. Pages 9-25 in Miranda M, ed. Surubim, Belo Horizonte: Instituto Brasileiro do Meio Ambiente e dos Recursos Naturais Renováveis.

Thorbjarnarson J, Wang X, He L. 2001. Reproductive ecology of the Chinese alligator (Alligator sinensis) and implications for conservation. Journal of Herpetology 35:553-558.

Thorbjarnarson JB. 2010. Black caiman Melanosuchus niger. Pages 29-39. in IUCN/SSC Crocodile Specialist Group, ed. Crocodiles: Status Survey and Conservation Action Plan. IUCN.

Trewavas E. 1977. The sciaenid fishes (croakers or drums) of the Indo-West-Pacific. Transactions of the Zoological Society of London 33:253-541.

Vecsei P, Artyukhin E. 2001. Threatened fishes of the world: Acipenser persicus Borodin, 1897 (Acipenseridae). Environmental Biology of Fishes 61:160.

Walsh B. 1989. Aestivation in the Australian Freshwater Crocodile? Australian Zoologist 25:68-70.

Wang X, Zhang K, Wang Z, Ding Y, Wu W, Huang S. 2004. The decline of the Chinese giant salamander Andrias davidianus and implications for its conservation. Oryx 38:197-202.

Waqas U, Malik MI, Khokhar LA. 2012. Conservation of Indus River Dolphin (Platanista gangetica minor) in the Indus River system, Pakistan: an overview. Records: Zoological Survey of Pakistan 21:82-85.

Wintner SP, Dudley SFJ, Kistnasamy N, Everett B. 2002. Age and growth estimates for the Zambezi shark, Carcharhinus leucas, from the east coast of South Africa. Marine and Freshwater Research 53:557-566.

Yang F, Zhang Q, Xu Y, Jiang G, Wang Y, Wang D. 2008. Preliminary hazard assessment of polychlorinated biphenyls, polybrominated diphenyl ethers, and polychlorinated dibenzo-p-dioxins and dibenzofurans to Yangtze finless porpoise in Dongting Lake, China. Environmental Toxicology and Chemistry 27:991-996.

Zhang S. 2001. Fauna Sinica Osteichthyes: Acipenseriformes, Elopiformes, Clupeiformes, Gonorhynchiformes. Science Press.

Zhou K. 1986. A project to translocate the Baiji, Lipotes vexillifer, from the mainstream of the Yangtze River to Tongling Baiji Semi-nature Reserve. Aquatic Mammals 12:21-24.

Zhou K, Qian W, Li Y. 1977. Studies on the distribution of baiji, Lipotes vexillifer Miller. Acta Zoologica Sinica 23:72-79.
